# Caffeine-mediated CD39^+^ Treg activation via the CD39-adenosine receptor pathway is a novel risk factor for pulmonary tuberculosis

**DOI:** 10.3389/fimmu.2026.1784235

**Published:** 2026-04-20

**Authors:** Liangyu Zhu, Zida Zheng, Xun Huang, Haoran Lu, Zhiqiang Chen, Hanxin Wu, Li Peng, Lvyan Tao, Yue Bai, Rui Yang, Ruian Bao, Suyi Luo, Weijiang Ma, Jieqin Song, Jiaomei Tang, Bingxue Li, Fukai Bao, Aihua Liu

**Affiliations:** 1 Department of Pathogen Biology and Immunology and Department of Biochemistry and Molecular Biology, Faculty of Basic Medical Sciences, Kunming Medical University, Kunming, China; 2 Department of Pathogen Biology and Immunology, Haiyuan College, Kunming Medical University, Kunming, China

**Keywords:** adenosine receptor, caffeine, eQTL, mendelian randomization, pulmonary tuberculosis, Tregs

## Abstract

**Background:**

The global impact of pulmonary tuberculosis (PTB) is compounded by a limited understanding of modifiable risk factors. While caffeine is the most consumed psychoactive substance, its causal relationship with PTB and the underlying immunological mechanisms remain unknown.

**Methods:**

A three-tiered approach was used: 1) two-sample Mendelian randomization (TSMR) was used to analyze 486 metabolites and 731 immune cells for PTB causality (inverse variance weighting was the primary method with reverse MR and Bonferroni correction), 2) single-cell RNA sequencing (scRNA-seq) and bulk RNA-seq were integrated (Seurat, Gene set enrichment analysis, and pseudotime analysis) to characterize CD39^+^ Tregs traits in lungs with PTB using, 3) core genes (LASSO) regression and eQTL-based genetic analyses uniquely) were validated in THP-1 macrophages, C3HeB/FeJ mice, and patients with PTB via FCM, WB, RT-qPCR, and multiplex immunohistochemistry.

**Results:**

Based on TSMR, eight metabolites (including caffeine) and nine immune subsets (including activated CD4^+^ Tregs) were linked to PTB (*P* < 0.05). Caffeine increased the risk of PTB via CD39^+^CD4^+^ Tregs (mediated proportion = 10.4%, *P* = 0.046). ScRNA-seq analysis of PTB lungs revealed elevated CD4^+^ Tregs with high caffeine responsiveness and CD39/ADORA2A overexpression. Validation in models revealed that core genes (PSMC5, BAG1, and AGPAT5) exhibited differential expression with PTB (*P* < 0.05) and good diagnostic efficacy (AUC > 0.7).

**Conclusions:**

We first identified a causal association between genetically predicted caffeine levels and PTB risk at the genetic level. We further uncovered a CD39-adenosine-based Treg activation mechanism underlying this association, and identified PSMC5 as a potential therapeutic target for host-directed therapy. These findings inform PTB pathogenesis and host-directed therapy.

## 1 Introduction

Tuberculosis (TB), which is caused by *Mycobacterium tuberculosis* (Mtb), is a major global health threat, and pulmonary TB (PTB) accounts for approximately 80% of all TB cases (1). In 2024, there were 10.7 million new cases of TB worldwide, resulting in 1.2 million deaths (1). Despite advances in TB treatment, the emergence of multidrug-resistant strains and the limited protective efficacy of existing vaccines underscore the urgent need to identify environmental factors that modulate host susceptibility (2, 3). Caffeine levels in the body have been demonstrated to exert complex regulatory effects on the immune system (4, 5). Notably, epidemiological and Mendelian randomization (MR) studies have demonstrated that caffeine and coffee intake long-term heavy consumption is associated with adverse lipid profiles including higher low-density lipoprotein cholesterol, increased risk of cardiovascular disease (6), and elevated risks of osteoarthrosis, arthropathy and obesity (7). However, its functional role in infectious diseases, particularly pulmonary tuberculosis, remains to be elucidated.

Regulatory T cells (Tregs) play a dual regulatory role in Mtb infection. Although moderate Treg expansion effectively prevents immunopathological damage, excessive activation suppresses protective Th1 immune responses, promoting PTB development (8). The core mechanism of Treg-mediated immunosuppression relies on the CD39 (ectonucleoside triphosphate diphosphohydrolase 1 [ENTPD1])–adenosine pathway, whereby CD39 catalyzes extracellular ATP hydrolysis into adenosine while activating the immunosuppressive adenosine A2A receptor (A2A-R) (9). Moreover, recent studies suggest that caffeine can enhance Treg activity *in vivo* through an unknown mechanism (10, 11).

There are key knowledge gaps about the role of caffeine in PTB immune regulation. First, lifestyle factors like smoking and diet confound studies linking caffeine to infection risk, making it difficult to establish a causal relationship between caffeine and PTB (12). By leveraging Mendelian inheritance patterns and employing genetic variants as instrumental variables (IVs) to simulate randomized controlled trials, MR offers a reliable method for deciphering causal associations between PTB risk factors (13). The functional heterogeneity of caffeine-responsive Treg subsets and their specific roles in PTB pathogenesis are unclear. Although CD39 plays a central role in adenosine-mediated immunosuppression, the differentiation pathways, molecular characteristics, and caffeine responsiveness of CD39^+^ Tregs in PTB lung tissue require further investigation (14). Although host-directed therapies for diseases like sepsis (15) and ovarian cancer (16) have utilized the CD39–A2A-R pathway, there is a gap in its clinical value for PTB. The identification of the core genes associated with this pathway and their drug targets can provide a new approach for host-guided PTB immunotherapy.

**Figure 1:**
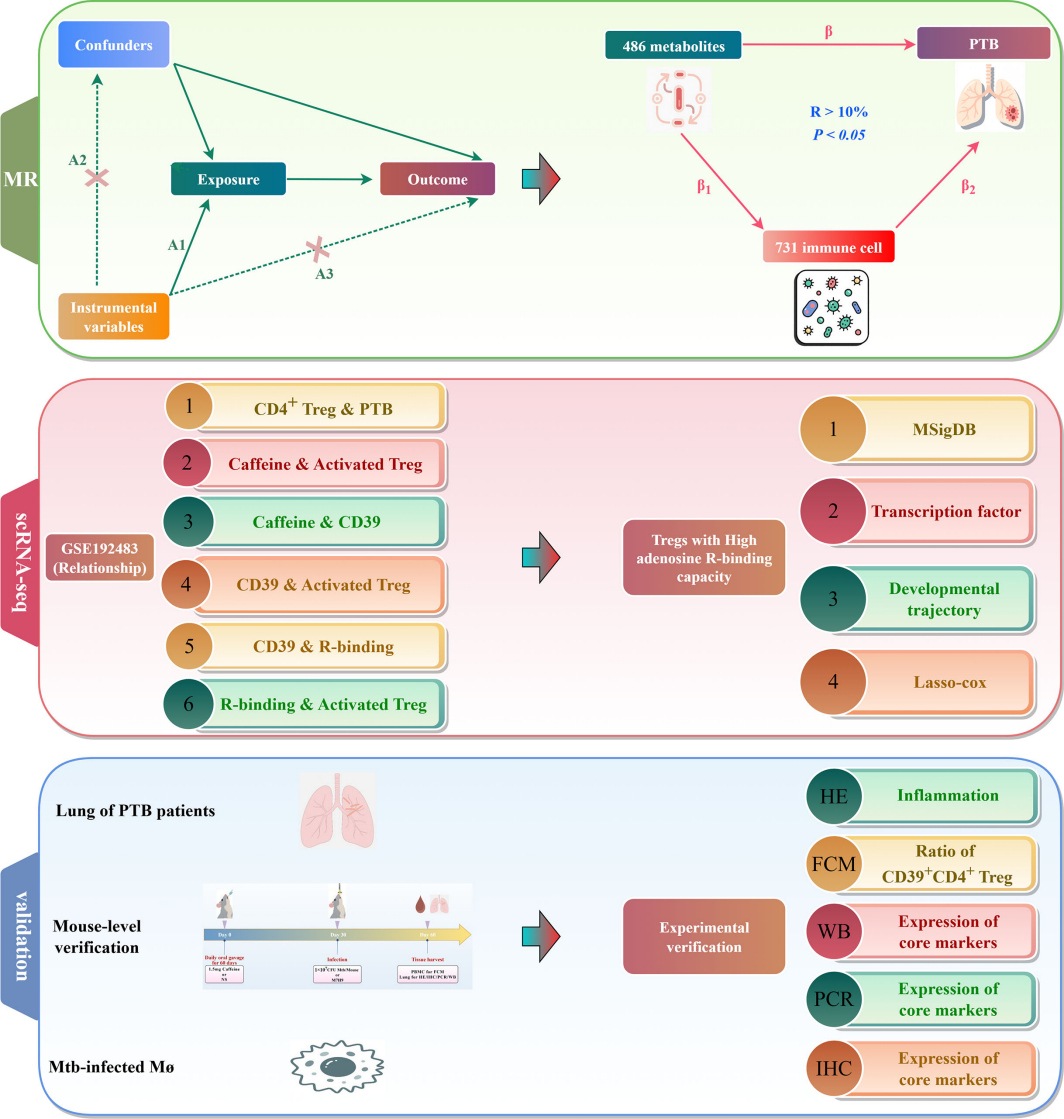
Study flow chart. This study elucidated the mechanism by which the caffeine–CD4^+^CD39^+^ Treg pathway regulates PTB through causal inference (based on MR), integration of single-cell and bulk RNA-seq analysis, and experimental validation at the cellular, animal, and human levels. A1 (IVs relevance [IVs are associated with exposure]). A2 (IVs independence [IVs are not associated with confounders]). A3 (no direct effect [IVs affect the risk of PTB via exposure, not through other pathways]). ScRNA-seq, single-cell RNA sequencing; FCM, flow cytometry; WB, western blot; PCR, polymerase chain reaction; IHC, immunohistochemistry; Mø, macrophage.

To address this gap, we employed two-sample Mendelian randomization (TSMR) to investigate a causal link between caffeine, CD39^+^ Tregs, and PTB risk based on genetic variation, thereby mitigating confounding factors. Single-cell RNA sequencing (scRNA-seq) of PTB lung tissue was used to determine the differentiation trajectory of CD39^+^ Tregs, their responsiveness to caffeine, and the activation characteristics of adenosine receptor pathways. Bulk RNA-seq, scRNA-seq, and MR data were integrated to screen for PTB-associated core genes. The findings were experimentally validated at the cellular, animal, and human levels. **Figure 1** shows the study’s overall roadmap.

## 2 Materials and methods

### 2.1 Study design

This study involved a three-part research framework consisting of “causal inference”, “single-cell analysis”, and “mechanism validation” (**Figure 1**). First, we screened 486 blood metabolites for causal associations with PTB using MR analysis and then analyzed the mediating effects of these metabolites using 731 immune cell eQTL data. Next, we used scRNA-seq and RNA-seq data from the lungs of patients with PTB, along with pseudotime series trajectory and transcriptional regulation analysis to elucidate the molecular mechanism by which caffeine activates CD39^+^ Tregs. A mouse model of TB infection was developed for experimental validation, with the caffeine and physiological saline groups being established simultaneously. Successful model development was confirmed through hematoxylin and eosin and acid-fast staining of lung tissues. The activation levels of CD4^+^CD25^+^FOXP3^+^ T cells were determined via flow cytometry. The expression changes of core genes, as well as proinflammatory and anti-inflammatory factors, were evaluated using western blot, RT-qPCR, and multiplex immunohistochemistry (mIHC). All data sources are summarized in **Table 1**.

**Table 1:** Details of the datasets included in this study.

Datasets	Source	Characteristic
PTB GWAS-1	AB1_RESP_TUBERCU_CONF (https://storage.googleapis.com/finngen-public-data-r8)	490 samples; respiratory TB was bacteriologically and histologically confirmed
PTB GWAS-2	finn-b-TBC_RESP (https://opengwas.io/datasets/finn-b-TBC_RESP)	849 cases; respiratory tuberculosis; European
PTB GWAS-3	finn-b-AB1_RESP_TUBERCU (https://opengwas.io/datasets/finn-b-AB1_RESP_TUBERCU)	849 cases; respiratory tuberculosis; European
PTB GWAS-4	finn-b-AB1_TUBERCULOSIS (https://opengwas.io/datasets/finn-b-AB1_TUBERCULOSIS)	1193 cases; tuberculosis; European
Caffeine GWAS	GCST90200436 (http://ftp.ebi.ac.uk/pub/databases/gwas/summary_statistics)	8005 samples; blood level
Activated & secreting CD4^+^ Tregs GWAS	GCST90001503 (http://ftp.ebi.ac.uk/pub/databases/gwas/summary_statistics)	3437 samples; blood level
39^+^73^-^CD4^+^ Tregs GWAS	met-b-189 (https://gwas.mrcieu.ac.uk/datasets/)	497 samples; PBMC
39^+^73^+^CD4^+^ Tregs GWAS	met-b-194 (https://gwas.mrcieu.ac.uk/datasets /)	497 samples; PBMC
39^+^CD4^+^ Tregs GWAS	met-b-193 (https://gwas.mrcieu.ac.uk/datasets/)	497 samples; PBMC
39^-^73^+^CD4^+^ Tregs GWAS	met-b-192 (https://gwas.mrcieu.ac.uk/datasets/)	497 samples; PBMC
73^+^CD4^+^ Tregs GWAS	met-b-193 (https://gwas.mrcieu.ac.uk/datasets/ )	497 samples; PBMC
PSMC5 eQTL GWAS	eqtl-a-ENSG00000087191 (https://opengwas.io/datasets/eqtl-a-ENSG00000087191)	14263 samples; European
PTB scRNA-seq	GSE192483 (https://www.ncbi.nlm.nih.gov/geo/query/acc.cgi)	11 PTB samples; lung tissues; sputum continuous positive or repeated positive localized lesions, and tuberculous pathological lesions
PTB bulk-1	GSE148036 (https://www.ncbi.nlm.nih.gov/geo/query/acc.cgi)	10 samples; lung tissues; culture-confirmed Mtb in sputum or bronchoalveolar lavage
PTB bulk-2	GSE114911 (https://www.ncbi.nlm.nih.gov/geo/query/acc.cgi)	36 samples; lung tissues; H37Rv strain

### 2.2 MR, colocalization, and regional association analysis

First, to exclude SNPs in linkage disequilibrium (LD), we applied criteria for selecting IVs with an LD coefficient *r*² < 0.001 and a clumping window size of 10,000 kb, thus ensuring the independence of IVs. Subsequently, we computed *R*² and F-statistics for each SNP and retained those with *F* > 10 to mitigate bias from weak instruments. Finally, palindromic SNPs were excluded if their minor allele frequency (MAF) was < 0.01, yielding the final set of IVs. TSMR was performed using the “Two Sample MR” R package to evaluate potential associations between blood metabolites and PTB. Causal effects were assessed using the IVW method, MR-Egger regression, and the weighted median estimator. IVW was used as the primary approach because of its higher statistical power. Reverse MR analysis was conducted to eliminate reverse causality. FDR correction was applied to avoid cumulative false positives from multiple testing. TSMR was used to evaluate the mediating effect of immune cells as confounders, and β values were converted to ORs with 95% confidence intervals.

For colocalization analysis, we applied the coloc.abf function in coloc (version 2.1.1) to calculate posterior probabilities for five hypotheses (H0–H4), which evaluate the likelihood of shared causal variants between PSMC5 eQTLs and PTB risk loci.

Regional association plots were generated using locuscomparer (version 1.0.2) to visualize overlapping genetic signals. We first identified SNPs common to both eQTL and GWAS datasets, then formatted these into input files.

### 2.3 Combined scRNA-seq and bulk RNA-seq analysis

Referring to the previous study (13), the Seurat (4.0.2) package on R was used to process scRNA-seq data. Quality control was performed by excluding cells with extreme transcriptional profiles (>90% of the reads mapping to a single gene, <200 detected genes, or a mitochondrial gene content of >10%). Feature selection using the variance stabilization transformation approach in Seurat identified the top 2000 most variable genes for principal component analysis. We obtained 67,671 high-quality single cells after strict quality control. Among them, 30,748 T cells were annotated for subcluster analysis, including 1,285 CD4^+^ Treg cells. For differential expression analysis of Treg subgroups, genes with adjusted *P-value* < 0.05 and |log_2_ fold change (log_2_FC)| > 0.25 were identified as statistically significant DEGs, and the results were visualized via dot plots.

The “FindClusters” function was used for clustering at a resolution of 0.2. The “FindAllMarkers” function was used to identify differentially expressed genes (DEGs) in each cluster using the default parameters for normalized gene expression data in the “Seurat” package. Following batch correction using the “Harmony” package in R, cell-type-specific biomarkers were applied to annotate clusters, and cell type proportions were calculated and evaluated based on the “reshape2” (version 1.4.4) and “ggplot2” (version 3.4.4) packages in R. Cell type annotation of T cell clusters was performed by integrating previously published evidence (17) with the marker gene profiles available in the Cell Marker database (18).

For single-cell functional analysis, pseudotime trajectory analysis focused on CD4^+^ Treg cells, using slingshot (version 2.8.1) and SingleCellExperiment (version 1.22.0). Switch gene analysis was performed to identify genes with dynamic expression during T cell differentiation, using GeneSwitches (version 1.0.0) and mixtools (version 2.0.0). The “find switch logistic fastglm function” was used to fit logistic regression models for each gene, evaluating how expression correlates with pseudotime. Cell-cell communication was analyzed using CellChat (version 1.1.3). Metabolic pathway activity was quantified using scMetabolism (version 1.0.1).

### 2.4 MSigDB, transcription factors, and developmental trajectory analysis

GSEA was performed using the “clusterProfiler” package to identify significantly enriched biological pathways in the MsigDB Hallmark gene sets. For the functional characterization of cellular caffeine responsiveness in Treg subpopulations, two independent MSigDB gene sets were used for cross-validation: GOBP_CELLULAR_RESPONSE_TO_CAFFEINE (labeled as “response to caffeine-1”) and GOBP_RESPONSE_TO_CAFFEINE (labeled as “response to caffeine-2”). DEGs between Tregs with high and low adenosine receptor aggregation capacity were identified using Seurat’s FindMarkers function. Gene symbols were converted to Entrez IDs using the org.Hs.eg.db database before GSEA. Transcription factor activity was inferred through single-cell regulatory network analysis in the DoRothEA database implemented using the VIPER algorithm. Cell developmental trajectories were reconstructed using pseudotime analysis of transcriptional dynamics across cellular states.

### 2.5 DEGs screening and ROC analysis

DEGs were identified from bulk RNA-seq datasets (GSE114911, GSE148036) using limma (FDR-adjusted *P*-values: < 0.05, |log_2_FC| > 0.25). Gene expression matrices were log_2_-transformed after normalization, and batch effects were corrected using ComBat. LASSO regression (“glmnet” package) with 10-fold cross-validation was used to select optimal gene signatures from the marker genes identified in the scRNA-seq analysis. Heatmaps and violin plots were generated using the “pheatmap” and “ggplot2” packages, respectively. The predictive performance of the selected genes was evaluated using ROC curve analysis, based on the training cohort (n = 10) and the validation cohort (n = 37).

### 2.6 *In vivo* animal experiments

**Figure 2:**
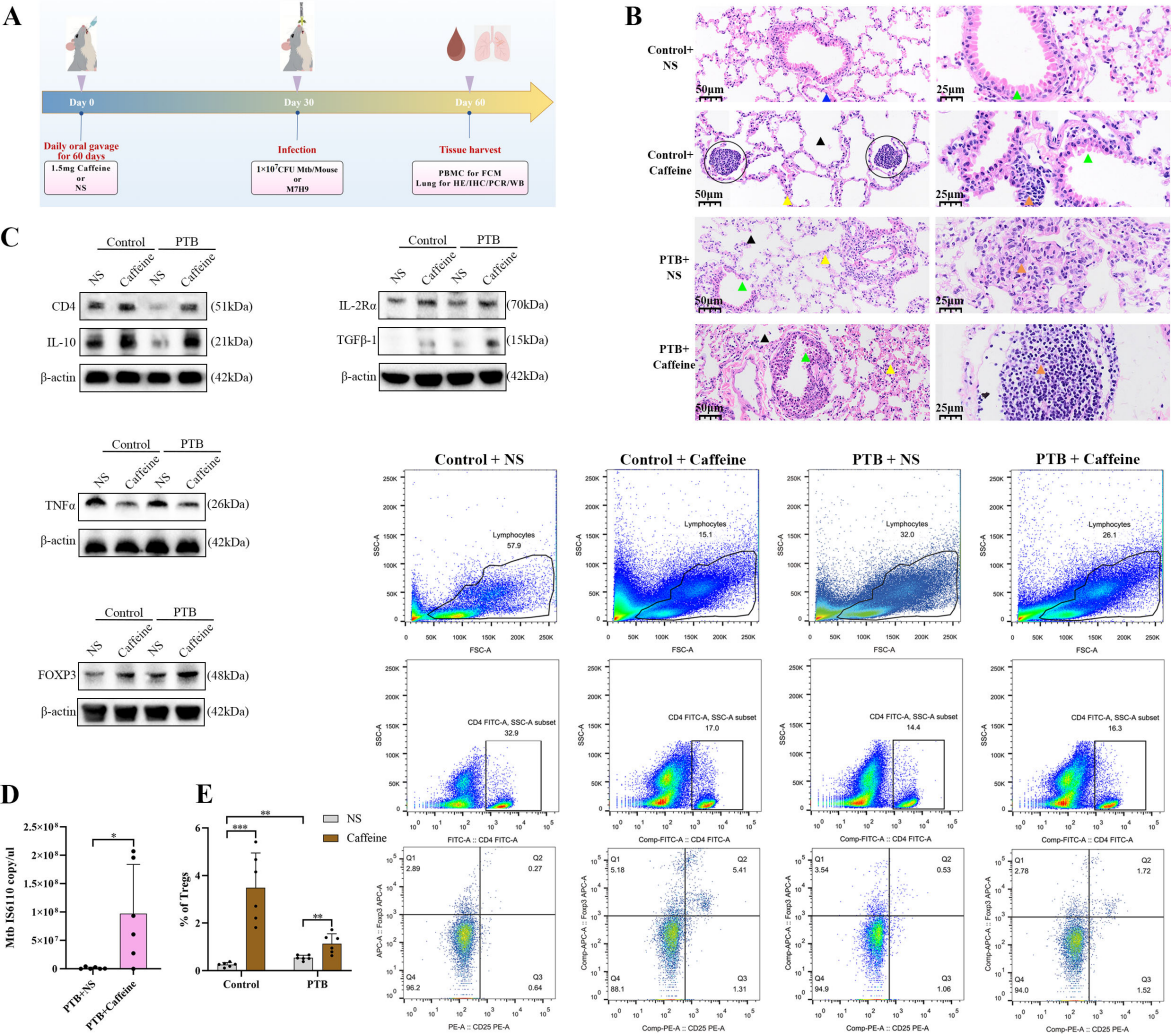
Caffeine promotes PTB by activating CD4^+^ Treg cells-mediated immunosuppression. **(A)** Schematic illustration of the experimental workflow, including caffeine administration by daily oral gavage for 60 days, Mtb infection, and tissue harvesting at designated time points. **(B)** H&E-stained lung sections from the control, control+caffeine, PTB (Mtb-infected), and PTB+caffeine groups, with black, green, blue, yellow, and orange arrows indicating the alveolar space, bronchus, vessel wall, alveolar septum, and lymphocytic infiltration, respectively. The black circle represents a lymphoid nodule. **(C)** WB analysis of target proteins in lung tissues from 4 experimental groups: FOXP3, TNF-α, CD4 and IL-10, IL-2Rα, TGF-β1, PSMC5, BAG1, and AGPAT5. **(D)** Absolute quantitative PCR was used to detect Mtb load in lung tissue. **(E)** FCM analysis of CD4^+^CD25^+^FOXP3^+^ Tregs in PBMC, with Treg quantification (left) and dot plots (right). Data are presented as mean ± SD (n = 6 biologically independent mice per group). ns, *, **, and *** indicate not significant, *P <* 0.05, *<* 0.01, and *<* 0.001.

C3HeB/FeJ mice were used to model PTB because of their high pathological similarity to humans (19). The mice were randomly assigned to the intratracheal M7H9 medium+oral saline gavage (vehicle), the intratracheal M7H9+oral 0.1% caffeine (1.5 mg/mouse daily for 60 days; caffeine control), the Mtb (1 × 10^7^ CFU/mouse) bronchial instillation+oral saline (infection control), and the Mtb instillation+oral caffeine (infection+caffeine) groups (6 per group). Treatment via oral gavage was commenced one month before infection and continued throughout the experimental period. Mouse chronic caffeine intake was used to simulate the usual dosage range in humans (20). (**Figure 2A**) shows a flowchart of the experiment.

### 2.7 *In vitro* cell experiments

THP-1 monocytes were differentiated into macrophages using 200 ng/mL phorbol 12-myristate 13-acetate for 72 hours. Differentiated macrophages were serum-starved for 12 hours before infection with Mtb (MOI: 10). The cells were washed 6 hours after infection to remove extracellular Mtb, followed by incubation for an additional 48 hours. Cells were harvested 48 hours post-infection.

### 2.8 Flow cytometry

Peripheral blood in EDTA-coated tubes was treated with erythrocyte lysis buffer (Beyotime, C3702) for five minutes at room temperature and then centrifuged at 1,500 rpm for five minutes. The resulting PBMCs were washed twice with PBS and stained with FITC-labeled anti-CD4 (Invitrogen, 11-0042-86) and PE-labeled anti-CD25 (Invitrogen, 12-0251-82) antibodies for 30 minutes at 4 °C in the dark. After washing, cells were fixed and permeabilized using a commercial kit (Invitrogen, GAS004) and then stained with FOXP3-APC (Invitrogen, 17-5773-82) for 45 minutes at 4 °C. The samples were analyzed on a BD FACSCanto II flow cytometer (excitation: 490 nm, 565 nm, and 650 nm) with a minimum acquisition of 10000 events per tube. CD4^+^CD25^+^FOXP3^+^ Treg populations were gated and quantified for mean fluorescence intensity using FlowJo v10.8.

### 2.9 RT-qPCR

Total RNA was isolated using TRIzol (Takara, 9108) and then reverse-transcribed into cDNA using a PrimeScript RT reagent kit (Takara, RR047A). RT-qPCR analysis was performed using TB Green Premix Ex Taq (Takara, RR047A) on a Bio-Rad CFX96 system and the following program: initial denaturation at 95 °C for 10 minutes, followed by 40 cycles of 98 °C for 30 seconds, 60 °C for 30 seconds, and 72 °C for 60 seconds, followed by a melt curve analysis at 65–97 °C to verify amplification specificity. β-actin was used as the reference gene, and relative gene expression was calculated using the 2^-ΔΔCT^ method. Primer sequences are listed in **Table 2**.

**Table 2:** PCR primer sequences.

Gene	Forward (5’–3’)	Reverse (5’–3’)
PSMC5 (human)	AGAATGGTGAGGGAGCTGTT	GTTGAGCAACTCCAGCATCG
BAG1 (human)	AAGATGGTTGCCGGGTCATG	TGTTCTGCTCCACTGTGTCAC
AGPAT5 (human)	CTGGTGCTCCACACGTACTC	CCAGGCCAACACGTAGGTG
β-actin (human)	CCTGGCACCCAGCACAAT	GGGCCGGACTCGTCATAC
PSMC5 (mouse)	GCCAGAATCTCCGTAGACTGC	CGACTTCTCCAACGTAGGAGC
BAG1 (mouse)	CCCACAGCAATGAGAGGTATGAC	TTGCAGGTGGTTAGCTATCTTCTC
AGPAT5 (mouse)	GGACATGTGCGCTACGTACT	AGATACATCGGTGTTCCTGCG
IL-10 (mouse)	GGCGCTGTCATCGATTTCTC	GCCTTGTAGACACCTTGGTCTTG
ENTPD1 (mouse)	TGGTGCAGCAGTTAGAGGAATG	CGCACCGATTTCATCTGTTTT
NT5E (mouse)	ATGAACATCCTGGGCTACGA	GTCCTTCCACACCGTTATCAA
ADORA1 (mouse)	TTCTCTCCCTTGTGGTAGGC	CAACACTGCCGTTGGCTATC
ADORA2A (mouse)	TGCAGAACGTCACCAACTTC	CAAAACAGGCGAAGAAGAGG
ADORA2B (mouse)	GCGTCCCGCTCAGGTATAAA	CAGTGGAGGAAGGACACACC
ADORA3 (mouse)	GTTCCGTGGTCAGTTTGGAT	GCGCAAACAAGAAGAGAACC
TNFa (mouse)	TCTTCTCATTCCTGCTTGTGG	ATGAGAGGGAGGCCATTTG
β-actin (mouse)	GTGCTATGTTGCTCTAGACTTCG	ATGCCACAGGATTCCATACC
IS6110 (Mtb)	ACCGAAGAATCCGCTGAGCT	GACGCGGTCTTTAAAATCGC

### 2.10 Western blot

Mouse lung tissues were ground through liquid nitrogen freeze-thaw cycles, homogenized in ice-cold RIPA lysis buffer containing protease inhibitors (PMSF), incubated on ice for 10 minutes, and then centrifuged at 12,000 g for 15 minutes at 4 °C. Protein concentration in the supernatant was determined using a BCA assay kit (TIANGEN, PA115). Next, protein samples (50 μg) were separated on 10% Novex™ Tris-Glycine gels and then transferred onto PVDF membranes (MERCK, HVLP14250). The membranes were then blocked using a serum-free blocking buffer for 30 minutes followed by overnight incubation at 4 °C with primary antibodies against β-actin (1:500000, Abclonal, AC026), PSMC5 (1:3000, Solarbio, K109756P), BAG1 (1:1500, FineTest, FNab00786), AGPAT5 (1:3000, CUSABIO, CSB-PA001), IL-10 (1:800, ABclonal, A2171), TNFα (1:3000, ABclonal, A23264), CD4 (1:1000, Abcam, ab288724), TGFβ1 (1:1000, ABclonal, A25313), FOXP3 (1:1000, Abcam, ab253297), and IL-2Rα (1:1000, Abcam, ab202911). The membranes were then washed and incubated with HRP-conjugated goat anti-rabbit IgG (1:15000, Servicebio, GB23303) for one hour at room temperature. Protein bands were then visualized using an ECL substrate (Biosharp, BL523A) and imaged using a Gel Doc XR^+^ imaging system. Relative band intensities were quantified using ImageJ with β-actin normalization.

### 2.11 MIHC

MIHC staining was performed using a cyclic tyramide signal amplification approach. Frozen lung tissues were sectioned at 4 μm, followed by endogenous peroxidase inactivation using 0.3% methanol–H_2_O_2_ for 15 minutes. The sections were then circled using a histochemical pen, blocked with 5% BSA at room temperature for one hour, and then incubated with the first primary antibody at 4 °C (overnight) in a humidified chamber. Next, they were incubated with an HRP-conjugated secondary antibody for 50 minutes at room temperature, and then with the tyramide signal amplification fluorescent dye including TYR-520 plus, TYR-570 plus, or TYR-690 plus (1:200, Y&K Bio, YK2330p) for 10 minutes in the dark. For the second and third fluorescent targets, antibodies were stripped with glycine–HCl (PH = 2.0) for 12 minutes, followed by endogenous peroxidase inactivation and blocking before incubation with the next set of primary and secondary antibodies. Finally, nuclei were counterstained with DAPI (MCE, HY-D-814) for 10 minutes. The sections were imaged on an Olympus FV3000 confocal microscope.

### 2.12 Human tissue samples and ethics

Human lung tissue samples were obtained from patients with PTB who underwent therapeutic surgical resection at The First Affiliated Hospital of Kunming Medical University. All samples were excess tissue after routine pathological diagnosis. Clinical PTB samples were confirmed by acid-fast staining and Mtb-specific PCR prior to use in this study. The study protocol was approved by the Medical Ethics Committee of Kunming Medical University (KMMU2024MEC276). Prior written informed consent was obtained from all patients, which included permission for the use of tissue in molecular research and the publication of findings. Inclusion criteria for healthy controls (1): Negative for Mtb infection via acid-fast staining, Mtb-specific PCR, and T-SPOT.TB assay (2); No history of tuberculosis or anti-tuberculosis treatment (3); No chronic inflammatory lung disease, autoimmune disease, or malignant tumor (4); No systemic immunosuppressive agent or glucocorticoid use within 6 months prior to surgery. Exclusion criteria for healthy controls (1): Any positive result for Mtb infection (2); History of active infectious disease within 4 weeks prior to surgery; (3) Severe dysfunction of vital organs.

### 2.13 Statistics

Mouse experiments were conducted with a minimum of 6 biological replicates (n=6), whereas cell-based experiments were carried out using no fewer than 3 biological replicates (n=3). Across all experimental groups, data are presented as the mean ± standard deviation (SD). For comparisons between two groups, either a t-test or non-parametric test was utilized. The latter was adopted when the assumption of a normal distribution was not tenable. Unless otherwise specified, all assessments of statistical significance were calculated relative to the reference control group, with the results presented accordingly. GraphPad Prism 8 software was employed for both data visualization and statistical analysis.

## 3 Results

### 3.1 Caffeine promotes PTB by activating CD39^+^CD4^+^ Tregs

**Figure 3:**
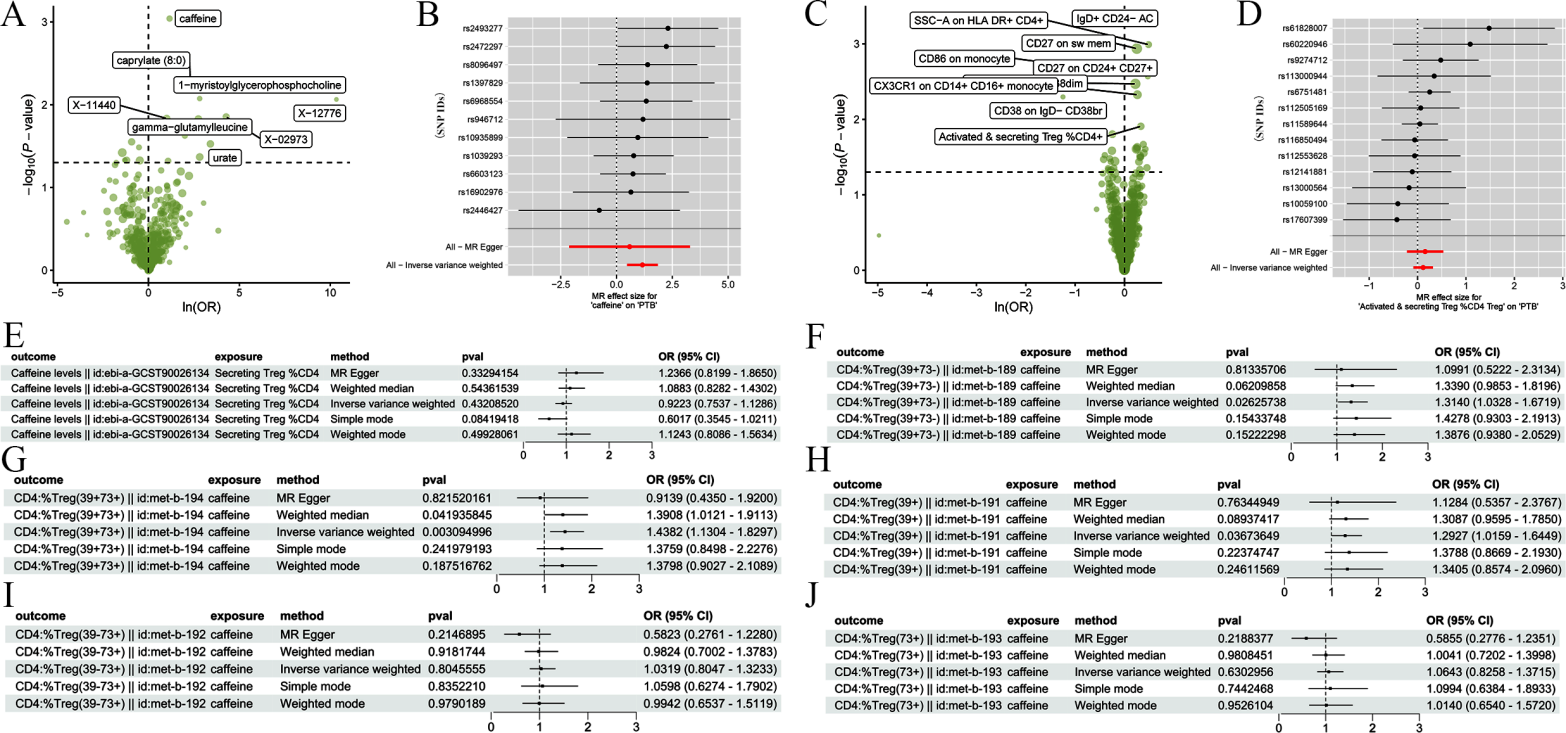
Mediated MR analysis revealed that caffeine promotes PTB by activating CD39^+^CD4^+^ Tregs. **(A, B)** Volcano **(A)** and forest plots **(B)** of the differential analysis of metabolites to identify key PTB-associated features (-log_10_ *P*-value vs ln [OR]). Y-axis of forest plots: reference SNP ID of qualified IVs for the 8 PTB-associated metabolites. Each row represents the causal effect estimate of a single SNP IV. **(C, D)** Volcano **(C)** and forest plots **(D)** of the differential analysis of immune cells to identify key PTB-associated features (-log_10_ *P*-value vs ln [OR]). Y-axis of forest plots: rs ID of qualified IVs (SNPs) for the 9 PTB-associated immune cell subsets. Each row represents the causal effect estimate of a single SNP IV. **(E)** A forest plot of five MR model methods verifying that secreting CD4^+^ Tregs mediate PTB risk. **(F)** A forest plot of five MR model methods verifying that caffeine mediates PTB risk. **(G–J)** Forest plots of five MR models verifying that caffeine mediates CD39^+^CD73^+^CD4^+^ Tregs **(G)**, CD39^+^CD4^+^ Tregs **(H)**, CD39^-^CD73^+^CD4 Tregs **(I)**, and CD73^+^CD4^+^ Tregs **(J)** risk. OR, odds ratio; CI, confidence interval.

A systematic assessment of the causal relationship between 486 blood metabolites and the risk of PTB using TSMR combined with inverse variance weighting (IVW) identified eight blood metabolites (caffeine, caprylate [8:0], 1-myristoylglycerophosphocholine, gamma-glutamylleucine, X-11440, X-02973, X-12776, and urate) as being significantly associated with PTB incidence (*P =* 9.092 × 10^−4^; (**Figures 3A, B**; **Supplementary Table 1**). To further elucidate the molecular mechanisms of PTB immune regulation, we assessed the causal relationship between 731 immune cell features and PTB risk. Through a TSMR approach, we identified nine immune cell subpopulations (SsC-A on HLA DR^+^CD4^+^, IgD^+^CD24^-^ AC, CD27 on SW mem, CD86 on monocyte, CD27 on CD24^+^CD27^+^, CX3CR1 on CD14^+^CD16^+^ monocyte, CD38 on IgD^-^CD38br, CD28^+^CD45RA^+^CD8dim%CD8dim, and Activated & secreting Tregs %CD4^+^) with significant causal associations (*P <* 0.05; **Figures 3C, D**; **Supplementary Table 2**). After adjusting the results for the FDR-corrected threshold, genetically anticipated Caffeine and activated CD4^+^ Tregs remained significantly linked to PTB risk (FDR < 0.01). Sensitivity analysis using the leave-one-out method confirmed that no single SNP significantly affected effect estimates (Δβ < 0.15), indicating a robust causal relationship (Cochran’s Q test: *P* > 0.05; MR–Egger regression intercept; *P* > 0.05).

Next, we assessed whether a mediating regulatory relationship between caffeine and CD4^+^ Tregs influences PTB risk. The results revealed positive associations between caffeine and PTB (odds ratio [OR] > 1, β = 1.162), caffeine and CD4^+^ Tregs (OR > 1, β_1_ = 0.363), and CD4^+^ Tregs and PTB (OR > 1, β_2_ = 0.331). The overall effect size of exposure on the outcome (β_all_ = 1.162) aligned with the direction of the mediating effect size (β_12_ = 0.120) in the exposure–outcome relationship, with a mediated proportion of 10.4% (*P* = 0.046; **Supplementary Table 3**). This suggests that caffeine exerts its mediating effect by influencing CD4^+^ Tregs. In addition, we tested all PTB-associated immune cells (monocytes, B cells included) and metabolites via 5 MR methods; only CD4^+^ Tregs (especially CD39^+^CD4^+^ Tregs) showed consistent significant mediation, so we focused on the caffeine-CD4^+^ Treg-PTB pathway as our core finding.

Reverse MR analysis excluded a causal effect of CD4^+^ Tregs on caffeine metabolism, confirming unidirectional causality (**Figure 3E**). CD39 and CD73 constitute the key pathway for extracellular adenosine production in Tregs (21). We therefore classified CD4^+^ Tregs into the CD39^+^CD73^-^, CD39^+^CD73^+^, CD39^-^CD73^+^, CD39^+^, and CD73^+^ subtypes. TSMR analysis of each subtype revealed that caffeine was significantly associated with increased risk in CD39^+^ Tregs only. Thus, the MR analysis results indicate that the CD39^+^CD4^+^ Treg subset is the main mediator of the increased risk of PTB onset associated with caffeine (*P* < 0.05; FDR < 0.01; (**Figures 3F–J**).

### 3.2 Lung tissues from patients with PTB exhibit significant CD4^+^ Treg activation and higher sensitivity to caffeine

**Figure 4:**
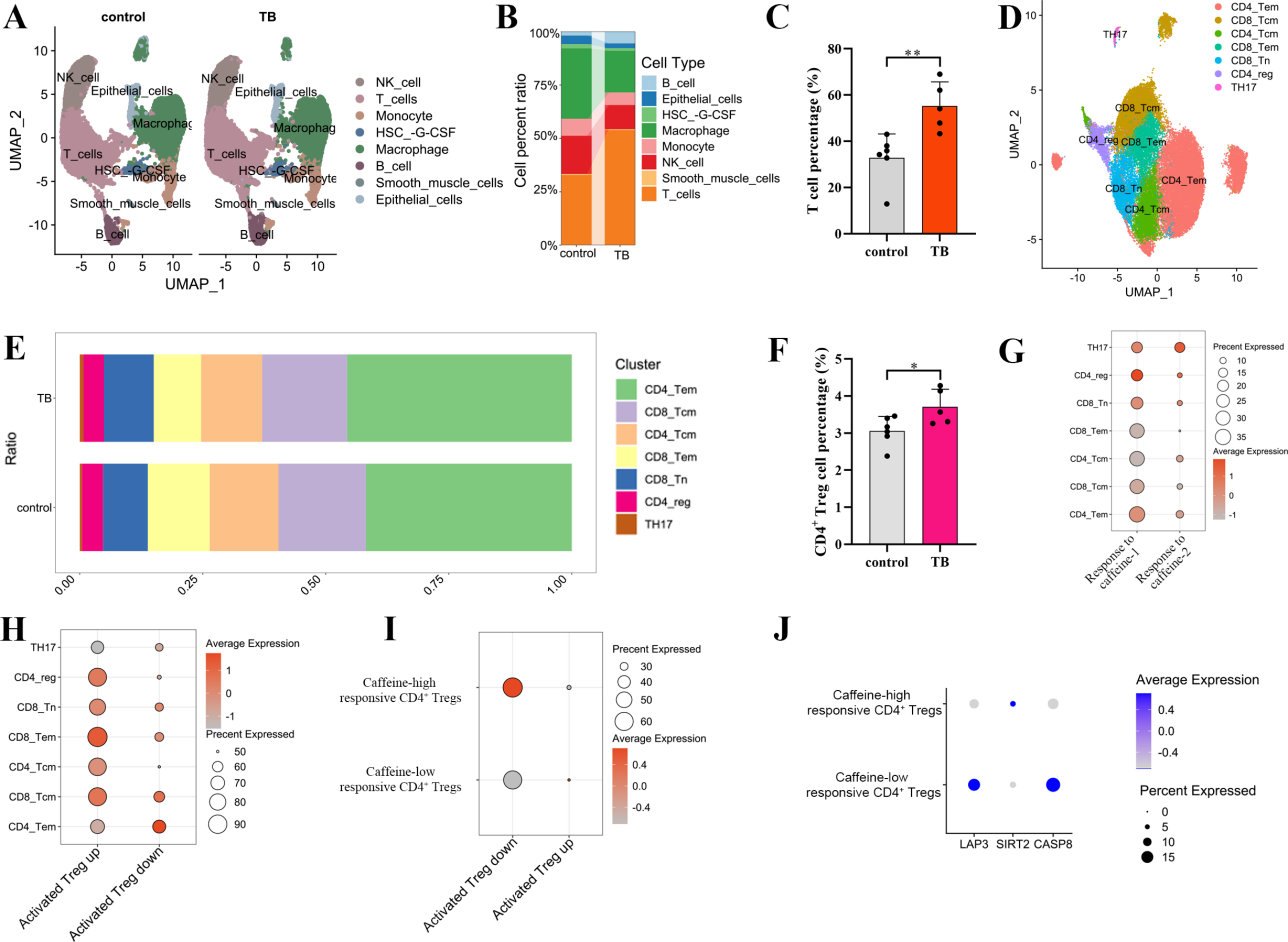
CD4^+^ Tregs from the lung tissues of patients with PTB were significantly activated and more sensitive to caffeine. **(A)** Single-cell UMAP clustering of lung tissues from the healthy and PTB groups. **(B)** Cell type proportions in both groups. **(C)** Quantification of the percentage of total T cells in control and TB groups. **(D)** UMAP clustering of identified T cell subsets. **(E)** Subset proportion distribution between groups. **(F)** Quantification of the percentage of CD4^+^ Treg cells in control and TB groups. **(G)** Expression of the gene sets associated with cellular response to caffeine, and activated Tregs in the T cell subsets. **(H–J)** Analysis of the expression of the gene set associated with activated Tregs in the high or low caffeine response CD4^+^ Tregs. In the dot plots, the color intensity represents the average expression level of the target gene, and the dot size indicates the percentage of cells expressing the gene within the CD4^+^ Treg subpopulation. UMAP: uniform manifold approximation and projection, NK_cell: Natural Killer Cell (9461 cells), T_cells: T Lymphocytes (30748 cells), Monocyte (4551 cells), HSC_-G-CSF: Hematopoietic Stem Cell Granulocyte Colony-Stimulating Factor mobilized (1037 cells), Macrophage (16837 cells), B_cell: B Lymphocytes (2752 cells), Smooth_muscle_cells: Smooth Muscle Cells (302 cells), Epithelial_cells: Epithelial Cells (1983 cells), TH17: T helper 17 cells (201 cells), CD4_reg: CD4^+^ Tregs (1285 cells), CD8_Tn: CD8^+^ naive T cells (3028 cells), CD8_Tem: CD8^+^ effector memory T cells (3216 cells), CD4_Tcm: CD4^+^ central memory T cells (3962 cells), CD8_Tcm: CD8^+^ central memory T cells (5353 cells), CD4_Tem: CD4^+^ effector memory T cells (13708 cells), Caffeine-high responsive CD4^+^ Tregs (642 cells), Caffeine-low responsive CD4^+^ Tregs (643 cells), CD39^+^ CD4^+^ Tregs (643 cells), CD39^-^ CD4^+^ Tregs (642 cells).

To validate the results of the MR analysis and explore the immune microenvironment of lung tissues with PTB, we performed scRNA-seq on lung tissue samples from patients with PTB. This analysis showed that when compared with healthy controls, lung tissues from patients with PTB had a significantly increased proportion of T cells (**Figures 4A–C**), with the CD4^+^ Tregs exhibiting a marked elevation among all T cell subsets (**Figures 4D–F**). This suggests that CD4^+^ Tregs may play a role in the regulation of host immune responses to Mtb through local immunosuppressive mechanisms. The number of all cell subtypes was listed in **Supplementary Table 4**. DEGs were listed in **Supplementary Table 5**. Further analysis revealed that CD4^+^ Tregs exhibited significantly higher sensitivity to caffeine stimulation (Molecular Signatures Database [MSigDB]: GOBP_CELLULAR_RESPONSE_TO_CAFFEINE, and GOBP_RESPONSE_TO_CAFFEINE) when compared with other immune cell subsets (**Figure 4G**; MSigDB: GSE15659_RESTING_VS_ACTIVATED_TREG_UP, and GSE15659_RESTING_VS_ACTIVATED_TREG_DN), and particularly, higher proportions were observed in activated Treg populations (**Figure 4H**; **Supplementary Tables 6**, **7**). Furthermore, based on differences in responsiveness to caffeine, we classified CD4^+^ Tregs into the high and low response subsets. The result showed that the high response Tregs exhibited significantly higher activation levels (**Figure 4I**). Gene expression profiling was used to determine the differential expression patterns of the genes associated with immune and inflammatory responses. The low caffeine-response Tregs showed significant enrichment of CASP8 associated with CD4^+^ Treg activation, while the high caffeine-response CD4^+^ Tregs exhibited significantly upregulated expression of the inflammation inhibitory gene SIRT2 (**Figure 4J**, adjusted *P* < 0.05, |log_2_FC| > 0.25). In summary, scRNA-seq analysis of lung tissue from patients with PTB validated the main findings of the MR analysis, i.e., lung tissue CD4^+^ Tregs are significantly activated and they exhibit higher sensitivity to caffeine.

### 3.3 Caffeine promotes PTB by activating CD4^+^ Treg cells-mediated immunosuppression

To clarify the role of caffeine in the pathogenesis of PTB under acute Mtb infection, animal experiments were conducted for verification (**Figure 2A**). Pulmonary histological analysis was performed to assess lung pathological changes in each group (**Figure 2B**). HE staining showed that the lung tissues of control mice had intact alveolar structure with no obvious inflammatory infiltration, while PTB+NS mice exhibited severe alveolar destruction, massive inflammatory cell aggregation, and extensive inflammatory lesion formation. Caffeine intervention markedly increased these infection-induced alveolar destruction and suppressed inflammatory infiltration in both healthy and PTB mice (**Figure 2B**). Quantitative pathological analysis further confirmed these findings: Mtb infection significantly increased pulmonary lymphocyte density and inflammatory lesion area fraction (both *P* < 0.01), while caffeine significantly suppressed both parameters in PTB and healthy mice (*P < 0.05 and **P < 0.01, respectively) (all ns, **Supplementary Figure 1**). WB results showed that Mtb infection downregulated the expression of Treg functional markers (FOXP3, IL-10, TGF-β1, IL-2Rα) and upregulated pro-inflammatory TNF-α in PTB mice (all *P* < 0.05). Caffeine intervention significantly restored Treg marker expression and suppressed TNF-α expression in PTB mice (all *P* < 0.05). No significant changes in the expression of inflammatory cytokines were observed in healthy control mice after caffeine treatment. (**Figure 2C**; all *P >* 0.05; **Supplementary Figure 2**). In the PTB mouse, a higher load of Mtb was detected in the lung tissues of mice treated with caffeine (**Figure 2D**; *P <* 0.05). PBMC flow cytometry revealed that the proportion of CD4^+^CD25^+^FOXP3^+^ T cells was significantly higher in the PTB+NS group than in the control+NS group (**Figure 2E**; *P <* 0.01). Moreover, the proportion of Tregs was significantly higher in the PTB+caffeine group when compared with the PTB+NS group (**Figure 2E**; *P <* 0.05).

### 3.4 Caffeine activates CD39^+^ Tregs by enhancing CD39-mediated adenosine receptor binding capacity

**Figure 5:**
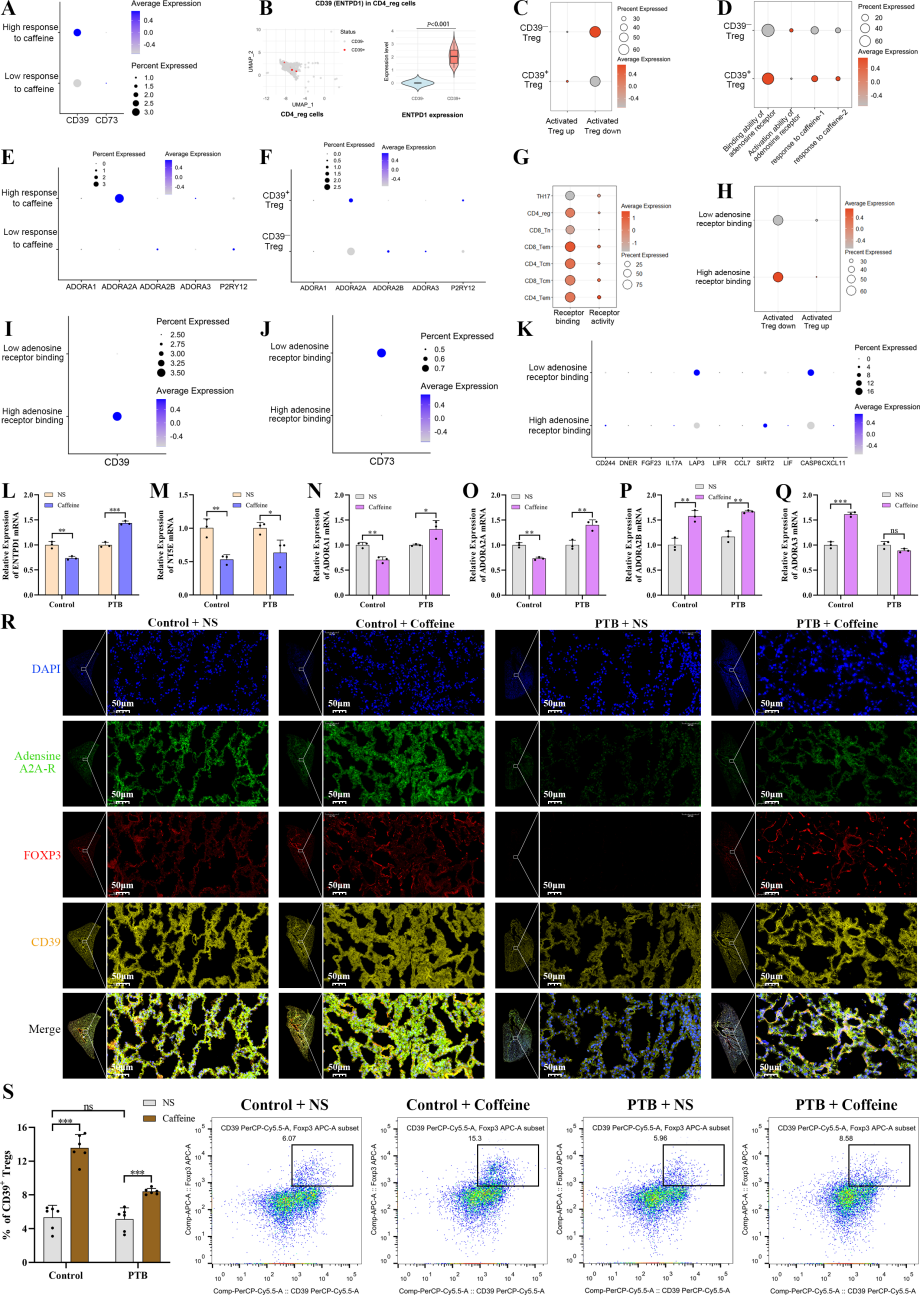
Caffeine activates CD39^+^ Tregs by enhancing the CD39-mediated adenosine receptor binding capacity. **(A)** Expression analysis of CD39 and CD73 in high and low caffeine response CD4^+^ Tregs. **(B–D)** Analysis of the expression of CD39 **(B)**, the gene set associated with activated Tregs **(C)**, and adenosine receptor binding capacity, adenosine receptor activation capacity, and two gene sets associated with cellular responses to caffeine in CD39-negative or positive CD4^+^ Tregs **(D)**. “response to caffeine-1” = MSigDB GOBP_CELLULAR_RESPONSE_TO_CAFFEINE; “response to caffeine-2” = MSigDB GOBP_RESPONSE_TO_CAFFEINE, two expert-curated gene sets used for cross-validation of cellular caffeine responsiveness. **(E, F)** Expression distribution of the genes associated with adenosine receptor activity in the CD4^+^ Tregs with high or low responsiveness to caffeine **(E)**, and in CD39^-^ or CD39^+^CD4^+^ Tregs **(F, G)** The expression distribution of the gene sets associated with adenosine receptor binding and activation capacity in all T cell subsets. **(H–K)** The expression distribution of the gene sets associated with Treg activation **(H, K)**, CD39 **(I)**, and CD73 **(J)** in CD4^+^ Tregs with high or low adenosine receptor binding capacity. **(L–Q**)RT-qPCR analysis of the relative expression levels of the key genes in lung tissues. **(R)** IF images of lung sections co-stained for A2A-R (green), FOXP3 (red), CD39 (yellow), and DAPI (blue [nuclei]). In the dot plots, the color intensity represents the average expression level of the target gene, and the dot size indicates the percentage of cells expressing the gene within the CD4^+^ Treg subpopulation. **(S)** FCM analysis of CD39^+^ in CD4^+^CD25^+^FOXP3^+^ Tregs of PBMC. Data are presented as mean ± SD. ns, *, **, and *** indicate not significant, *P <* 0.05, *<* 0.01, and *<* 0.001.

MR analysis found that caffeine specifically affects the CD39 receptor on CD4^+^ Tregs. To validate this finding, we conducted an in-depth analysis of the Treg subsets in lung tissues from patients with PTB using scRNA-seq and observed that CD39 (encoded by the ENTPD1 gene) was significantly upregulated in the high caffeine response CD4^+^ Tregs, which is consistent with the MR results. However, the expression of CD73 (which is encoded by the NT5E gene) was significantly higher in low caffeine response CD4^+^ Tregs (**Figure 5A**). Next, CD4^+^ Tregs were classified into the CD39^+^ and CD39^-^ subtypes (**Figure 5B**) combined visualization (FeaturePlot + violin plot). Expression analysis revealed that CD39 expression levels correlated positively with the Treg activation status and that CD39^-^ Tregs exhibited a low-activation state (**Figure 5C**). Further functional analysis revealed that compared with CD39^-^ Tregs, CD39^+^ Tregs had a significantly enhanced adenosine binding capacity (MSigDB: GOMF_ADENOSINE_RECEPTOR_BINDING, and GOMF_G_PROTEIN_COUPLED_ADENOSINE_RECEPTOR_ACTIVITY) and a markedly increased responsiveness to caffeine (both *P <* 0.05; (**Figure 5D**). Notably, we found synchronous transcriptional upregulation of A2A-R (encoded by the ADORA2A gene) in the high caffeine response and ENTPD1 (CD39-encoding)-high CD4^+^ Treg subsets (**Figures 5E, F**), suggesting that they share a synergistic regulatory mechanism in adenosine metabolic signaling pathways.

Moreover, we found that the expression of adenosine receptors (particularly ADORA2A) and their downstream activation-related genes was specific to the CD39^+^ Treg subset. Given CD39’s role in enhancing adenosine receptor binding capacity, we focused on the impact of adenosine receptor binding on CD4^+^ Tregs. First, we observed that adenosine receptor binding capacity significantly activated CD4^+^ Tregs (**Figures 5G, H**) and that it was associated with CD39^+^CD73^-^CD4^+^ Tregs (**Figures 5I, J**). Gene expression profiling showed that the adenosine receptor binding-competent subset was significantly enriched in the immunosuppression-related genes downregulated in activated Tregs (**Figure 5K**), which is consistent with caffeine (**Figure 4H**). This suggests that adenosine signaling may suppress CD4^+^ Tregs overactivation by regulating specific gene networks.

To explore the mechanism by which caffeine activates CD4^+^ Treg cells, we first identified that caffeine primarily activates CD39 rather than CD73 in the PTB model (**Figures 5L, M**; *P <* 0.001). Then, RT-qPCR revealed that when compared with the PTB+NS group, the expression of adenosine receptor (ADORA1, ADORA2A, and ADORA2B) was significantly increased following caffeine stimulation of caffeine (**Figures 5N–Q**; *P <* 0.001). To clarify the roles of CD39, A2A-R, and FOXP3 in caffeine-facilitated progression of PTB, we performed IF staining on lung tissues from normal control mice and PTB model mice before and after caffeine induction. MIHC staining showed that caffeine stimulation did not induce significant changes in the expression of CD39, Adenosine A2A-R and FOXP3 in the lung tissues of healthy control mice. In contrast, Mtb infection induced significant downregulation of these three markers from PTB model mice, and caffeine intervention notably reversed this infection-induced downregulation and restored the expression of CD39, Adenosine A2A-R and FOXP3 in Tregs (**Figure 5R**). Further colocalization analysis confirmed that more than 80% of CD39 and Adenosine A2A-R signals were specifically localized with FOXP3 in the PTB+Caffeine group, with a significant positive correlation between target protein signals and FOXP3 signals (**Supplementary Figure 3**). Flow cytometry data confirmed that caffeine significantly increased the proportion of CD39^+^ Tregs in the PBMC of Mtb-infected mice.(**Figure 5S**, **Supplementary Figure S4**). Collectively, these results indicate that the regulatory effects of caffeine on CD39, A2A-R, and FOXP3 expression are PTB pathological state-dependent. Caffeine may synergistically facilitate the pathological progression of PTB by activating the CD39-A2A-R adenosine signaling pathway and upregulating FOXP3 expression.

Our study indicated that the adenosine metabolism-related gene axis, ENTPD1–ADORA2A (CD39–A2A-R), is persistently activated in the high caffeine-responsive CD4^+^ Treg population. Specifically, CD39 initiates adenosine production by catalyzing ATP/ADP hydrolysis to AMP, while A2A-R-mediated adenosine signaling activates downstream immunosuppressive pathways through endocytosis. This “adenosine–A2A-R” cascade may maintain PTB microenvironment immune tolerance by modifying the functional state of Tregs.

### 3.5 High adenosine receptor binding capacity core markers were defined based on scRNA-seq combined with bulk RNA-seq

**Figure 6:**
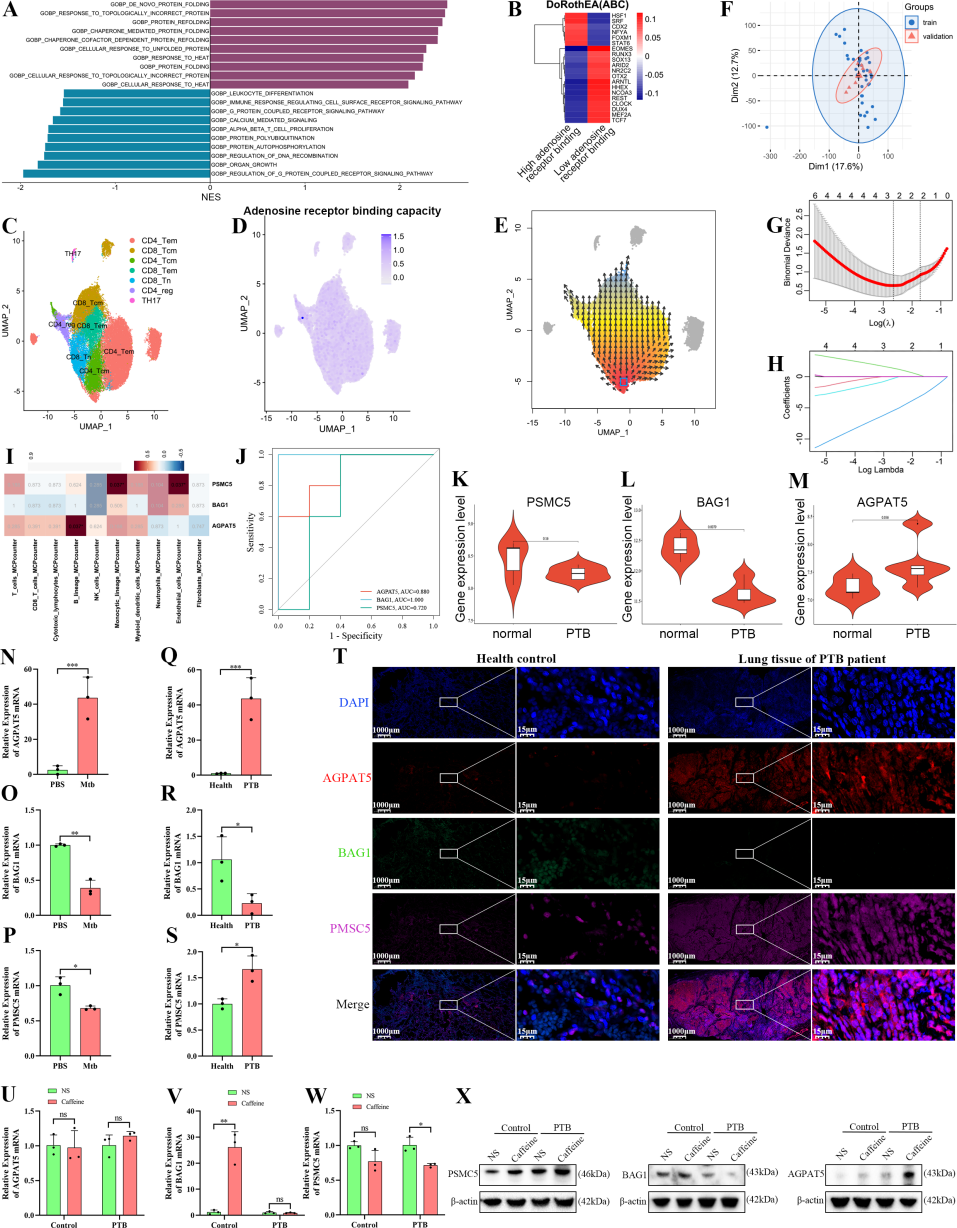
Identification of the core markers with high adenosine receptor binding capacity based on single-cell RNA-seq combined with bulk RNA-seq. **(A)** GSEA bar plot shows the significantly enriched pathways in Tregs with a high adenosine receptor binding. Different colors represent different pathways, while length indicates the significance of enrichment. **(B)** Heatmap visualization of the differences in the activity of key transcription factors across cell subsets (color gradient represents activity intensity). **(C–E)** Based on T cell subset clustering **(C)**, receptor binding feature visualization using UMAP **(D)**, and a developmental trajectory plot **(E)** show the single-cell distribution and developmental localization of the “Receptor binding1” feature (color gradient represents expression intensity). **(F)**Training and validation cohort distribution between two bulk RNA-seq datasets was visualized using dimension reduction plots. **(G, H)** Model fitting and optimization. A Bland–Altman plot **(G)** validating the consistency between two bulk RNA-seq datasets combined with LASSO regression **(H)** for dimensionality reduction of differential genes (curve intersections determine the optimal number of genes). **(I)** Heatmap visualization of correlations between core genes and immune phenotypes (color gradient represents correlation coefficients). **(J)** ROC curve validating the diagnostic efficacy for PTB. **(K–M)** Box plots of core gene (PSMC5, BAG1, AGPAT5) expression differences between normal and PTB tissues. **(N–R)** RT-qPCR analysis of the relative expression levels of the key genes in lung tissues. **(T)** IF images of lung sectionsfrom patients with confirmed PTB and healthy controls (age- and sex-matched individuals with no Mtb infection, histologically confirmed normal lung tissue), co-stained for AGPAT5 (red), BAG1 (green), PSMC5 (purple), and DAPI (blue [nuclei]). **(P–X)** RT-qPCR and Western blot analysis of the expression of core markers for CD4^+^ Treg cells adenosine receptor binding ability. Data are presented as mean ± SD. ns, *, **, and *** indicate not significant, *P <* 0.05, *<* 0.01, and *<* 0.001.

To determine the molecular characteristics of high adenosine receptor-binding CD4^+^ Tregs and their role in TB, single-cell combined with bulk RNA-seq sequencing data were analyzed. Gene set enrichment analysis (GSEA) revealed that processes like *de novo* protein folding, response to topologically incorrect proteins, cellular response to topologically incorrect proteins, and protein refolding were enhanced in high adenosine receptor-binding CD4^+^ Tregs, while leukocyte differentiation, the immune response-regulating cell surface receptor signaling pathway, and the protein-coupled receptor signaling pathway were attenuated (**Figure 6A**, **Supplementary Table 8**). In the CD4^+^ Tregs exhibiting high adenosine receptor binding scores, the activity of transcription factors like HSF1, SRF, and CDX2 was increased, while the activity of EOMES, RUNX3, and SOX13 was decreased (**Figure 6B**). These findings suggest a complex interplay between adenosine receptor binding levels, cellular function, and transcriptional regulation in PTB.

Next, we performed single-cell trajectory analysis to dissect immune cell dynamics in PTB. uniform manifold approximation and projection clustering (**Figure 6C**) partitioned immune cells into distinct subpopulations, including CD4^+^ Tem, CD8^+^ Tcm, and Th17. The “receptor binding” gene expression map (**Figure 6D**) showed a gradient distribution, with a markedly high expression in the CD4^+^ Treg cluster. Immune cell developmental hierarchy in PTB was clarified using pseudotime trajectory analysis (**Figure 6E**), which revealed that the cells originated from “progenitor-like” clusters and differentiated into mature subpopulations with a branch point indicating fate divergence. LASSO regression analysis was used to identify the core genes associated with high adenosine receptor binding capacity in PTB. To ensure data reliability, dimension reduction plots (**Figure 6F**) were used to visualize the distribution of the training and validation sets (GSE148036 and GSE114911, respectively). Gene selection was optimized using binomial deviance and cross-validation (**Figures 6G, H**). A heatmap visualization (**Figure 6I**) of the correlation scores between the identified core genes (PSMC5, BAG1, and AGPAT5) and immune cell infiltration highlighted their associations with immune responses in PTB. Validation of the diagnostic efficacy of these genes using ROC curve analysis (**Figure 6J**) indicated favorable specificity and sensitivity. Violin plot visualization of bulk RNA-seq data from PTB lung tissues *vs* normal samples (**Figures 6K–M**) revealed that BAG1, and AGPAT5 were differentially expressed, confirming their potential as PTB-related biomarkers.

**Figure 7:**
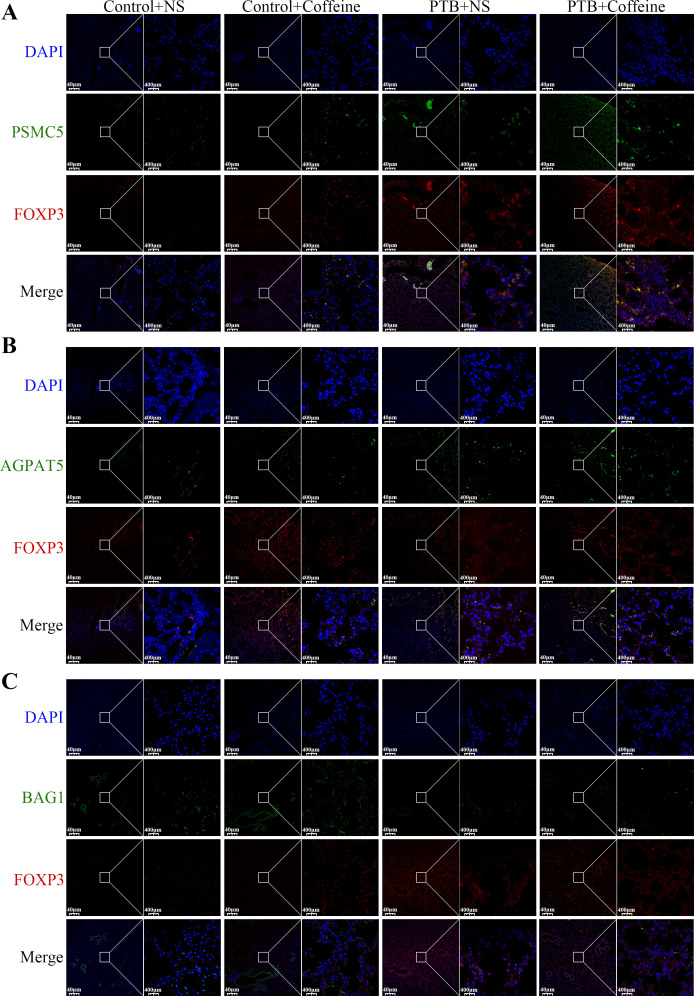
IF staining of PSMC5, AGPAT5, and BAG1 in FOXP3^+^ Tregs within mouse lung tissues. **(A–C)** IF images showing the expression and co-localization of PSMC5 **(A**, green), AGPAT5 **(B**, green), and BAG1 **(C**, green) with FOXP3^+^ Tregs (red) in lung tissues from four groups: Control+NS, Control+Caffeine, PTB+NS, and PTB+Caffeine. Nuclei were counterstained with DAPI (blue). Scale bar: 40 μm (left), 400 μm (right).

In experiments, we also found that key markers of adenosine receptor binding ability (AGPAT5, BAG1, and PSMC5) play an important role in tuberculosis. Specifically, AGPAT5 was significantly increased following Mtb stimulation of macrophages, while that of PSMC5 and BAG1 was significantly decreased (1–) (**Figures 6N–P**; all *P <* 0.05). When compared with the control+NS group, BAG1 expression was significantly reduced in the lung tissue of the mouse model of PTB, while that of PSMC5 and AGPAT5 was significantly increased (**Figures 6Q–S**, X; all *P <* 0.05). RT-qPCR revealed that PSMC5 mRNA was markedly downregulated by caffeine intervention in PTB mice (*P* < 0.05, **Figure 6W**). In contrast, WB showed the opposite trend (*P* < 0.05, **Figure 6X**). This transcriptional-translational uncoupling is may attributed to post-translational regulation of PSMC5 protein stability. Compared with healthy controls, the lung tissues of patients with PTB exhibited significantly higher AGPAT5 (red) and PSMC5 (purple), while BAG1 (green) expression was significantly suppressed (**Figure 6T**, all *P* < 0.001, **Supplementary Figure 5**). IF showed that caffeine significantly increased AGPAT5 and PSMC5 expression in control mice (all *P* < 0.05). In PTB mice, caffeine further upregulated AGPAT5, PSMC5, BAG1, and FOXP3 expression (**Figure 7**, all *P* < 0.05) and co-localization (**Supplementary Figure 6**, all *P* < 0.05). These results confirm that caffeine amplifies adenosine pathway markers expression in FOXP3^+^ Tregs during Mtb infection.

### 3.6 As a core marker of high adenosine receptor binding capacity, PSMC5 is a protective factor against PTB

**Figure 8:**
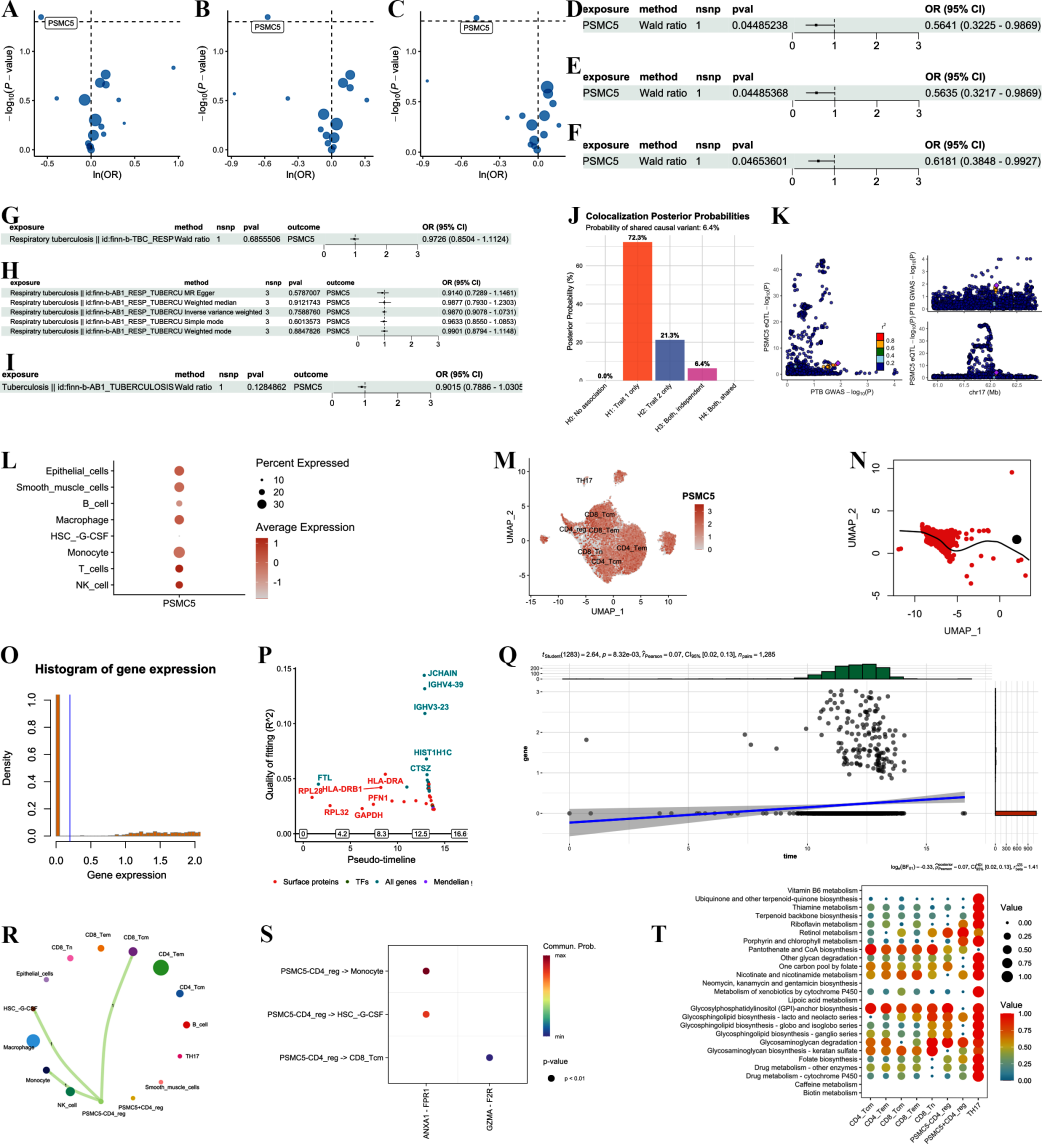
Multi-omics analyses of PSMC5 in PTB. **(A–C)** Volcano plots of MR effect estimates for eQTLs on three PTB outcomes (finn-b-TBC_RESP, finn-b-AB1_RESP_TUBERCU, finn-b-AB1_TUBERCULOSIS). X-axis: OR; Y-axis: outcome. Points = OR, error bars = 95% CI. **(D–F)** Forest plots for individual PSMC5 eQTLs (IVs). Rows = eQTLs; X-axis: OR for PTB risk. Dashed line = null effect (OR = 1). **(G–I)** Bidirectional MR plots for reverse causality. **(J)** Bar plot of colocalization posterior probabilities (H0–H4). H3 (shared causal variants: PSMC5 eQTLs vs. PTB GWAS) is labeled. **(K)** Regional association plot of PSMC5 locus (chr17). Blue = PSMC5 eQTLs, red = PTB GWAS SNPs; overlapping peaks highlighted. **(L, M)** PSMC5 expression patterns: **(L)** dot plot of PSMC5 expression across major lung cell types; **(M)** UMAP feature plot of single-cell PSMC5 distribution. **N** pseudotime trajectory UMAP overlaid with PSMC5 expression. **(O)** Density histogram of global PSMC5 expression distribution. **(Q)** Fitted dynamic expression trend of PSMC5 along pseudotime. **(P)** Scatter plot of pseudotime fitting quality (R²) for genes, transcription factors, surface proteins and Mendelian genes. **(Q)** Scatter plot of PSMC5 expression along pseudotime, with linear fitting curve (blue line, 95% CI in gray) and Pearson correlation statistics. **(R)** CellChat network plot. Nodes = cell types; edges = communication strength. PSMC5^+^CD4^+^ Treg cells are highlighted as central nodes. **(S)** Bubble plot of key ligand-receptor pathways for PSMC5^+^CD4^+^ Treg crosstalk. Size = communication count; color = ligand-receptor expression. **(T)** Heatmap of metabolic pathway activity in PSMC5^+^ vs. PSMC5^-^CD4^+^ Treg cells.

To elucidate the functions of the aforementioned PTB molecular targets (genes related to adenosine receptor binding capacity, AGPAT5, BAG1, PSMC5, CD4, IL-2RA, FOXP3, ENTPD1, NT5E, and FOXP3), eQTL (expression Quantitative Trait Loci), single-cell sequencing, and MR analyses were integrated at the genetic level. First, IVs reliability was ensured via pleiotropy/heterogeneity tests (MR-PRESSO, horizontal pleiotropy test, Cochran’s Q), which showed no strong evidence of horizontal pleiotropy or heterogeneity (*P* > 0.05). MR analyses showed only PSMC5 expression was associated with a protective effect on three PTB individuals, respectively finn-b-TBC_RESP (OR = 0.56; 95% CI: 0.32–0.99; *P* = 0.045; (**Figure 8A**), finn-b-AB1_RESP_TUBERCU (OR = 0.56; 95% CI: 0.32–0.99; *P* = 0.045; (**Figure 8B**), and finn-b-AB1_TUBERCULOSIS (OR = 0.62; 95% CI: 0.37–0.99; *P* = 0.047; (**Figure 8C**). Forest plots (all *P <* 0.05; (**Figures 8D–F**) visualized consistent protective effects across individual eQTLs. Bidirectional MR (PTB risk SNPs as exposures, PSMC5 expression as outcome) showed no significant reverse causation (all *P* > 0.05), ruling out reverse causality (**Figures 8G–I**). To verify shared genetic architecture, we performed colocalization on the PSMC5 gene locus (chr17) and PTB-associated GWAS signals. Colocalization analysis suggested that caffeine consumption and PTB risk are more likely regulated by distinct causal variants (H2, 21.3%) rather than sharing a common causal mechanism (H3, 6.4%, **Figure 8J**). Regional association plots (**Figure 8K**) illustrated overlapping significance peaks between PSMC5 eQTLs and PTB GWAS SNPs, reinforcing genetic linkage.

Then, PSMC5’s role in T cell differentiation, intercellular crosstalk, and metabolism were explored. Pseudotime trajectory analysis in CD4^+^ Treg cells showed PSMC5 expression dynamically increased along T cell activation/differentiation trajectories (**Figures 8L–O**), suggesting involvement in T cell functional maturation. Single-cell analyses revealed PSMC5’s functional relevance in T cells during PTB. To assess how gene expression correlates with immune cell differentiation, we quantified quality of fitting (R²) along a pseudo-timeline (**Figure 8P**). Genes associated with MR (purple, PSMC5) and surface proteins (red, e.g., HLA-DRB1, and HLA-DRA) exhibited weak R², indicating general correspondence between their expression and T cell differentiation. B cell- and macrophage-related genes (e.g., JCHAIN, IGHV, CTSZ) exhibit higher R² values, reflecting the cellular heterogeneity of the PTB lung immune microenvironment. Dynamic expression over pseudo-time (**Figure 8Q**) in a UMAP projection of CD4^+^ Treg cells, PSMC5 expression (black dots) increases significantly as pseudo-time progresses (linear regression: t-stat = 2.64, *P* = 8.39×10^-^³). This upward trend demonstrates PSMC5 is upregulated during T cell activation and differentiation in TB microenvironments, reinforcing its role in shaping T cell functional maturation.

Cell-cell interaction analysis was focused on PSMC5^+^CD4^+^ Treg cells in PTB lung tissues. The results showed that PSMC5^-^CD4^+^ Treg cells exhibited enriched signaling with monocytes, G-CSF-mobilized HSCs, and CD8^+^ central memory T cells (**Figure 8R**), particularly via ANXA1-FPR1 and GZMA-F2R pathways (**Figure 8S**), which are key mediators of anti-TB immunity. Metabolic pathway profiling revealed PSMC5^+^CD4^+^ Treg cells had enhanced activity in riboflavin metabolism, porphyrin and chlorophyll metabolism, and nicotinate and nicotinamide metabolism (**Figure 8T**), indicating metabolic reprogramming to support immune effector functions.

Collectively, multi-omics evidence demonstrates PSMC5 as a marker of high adenosine receptor binding capacity exerts a protective effect in PTB by shaping T cell differentiation, intercellular communication, and metabolic fitness.

## 4 Discussion

**Figure 9:**
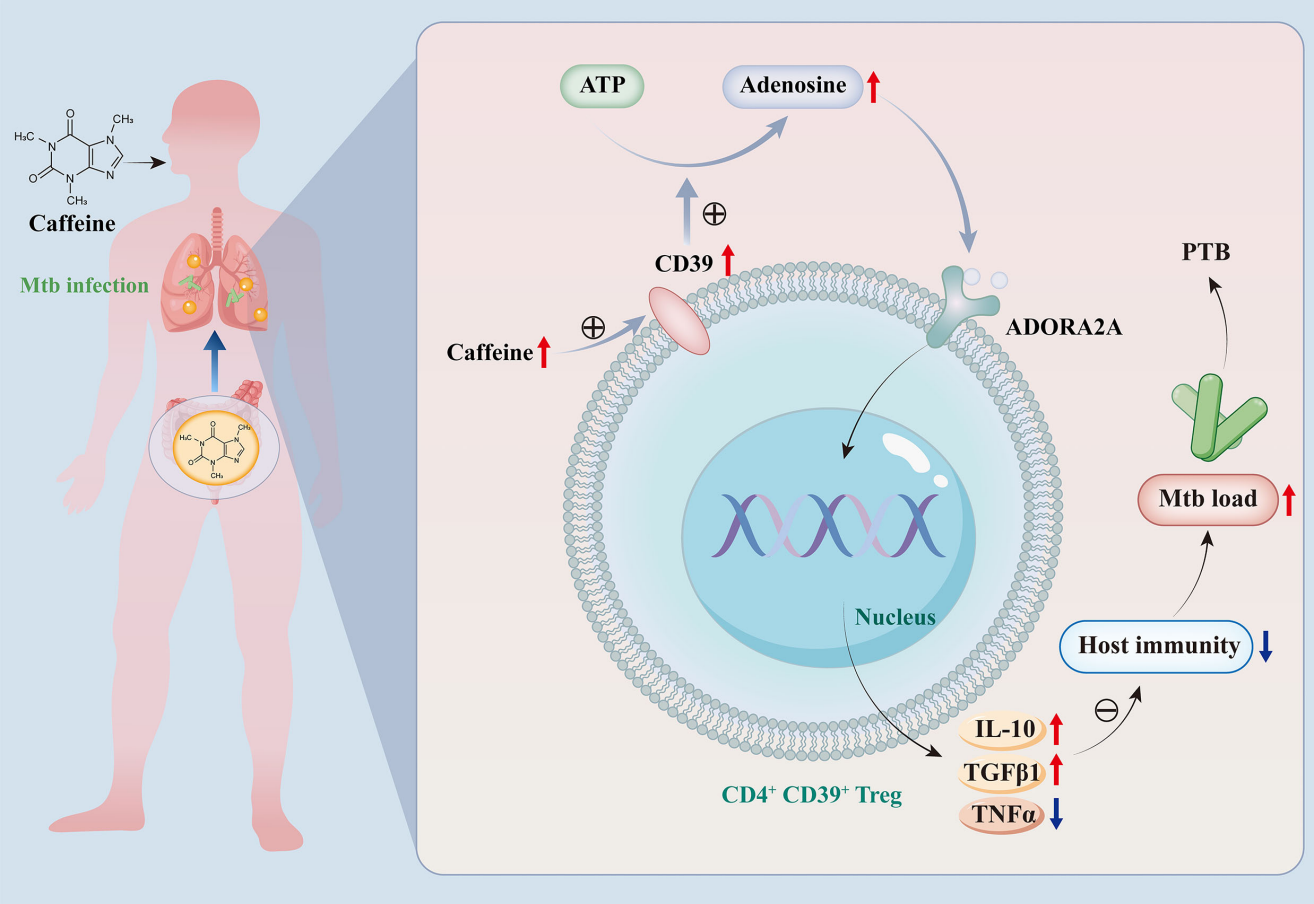
The mechanism by which caffeine modulates the risk of PTB via CD4^+^ Treg cells in patients with acute Mtb. Caffeine-activated CD4^+^ Tregs upregulate CD39, which catalyzes extracellular ATP hydrolysis to adenosine. AGPAT5, PSMC5, and BAG1 were identified as being associated with adenosine receptor binding capacity in PTB. Elevated levels of adenosine bind to the adenosine A2A-R on CD4^+^ Tregs. This binding activates downstream signaling, leading to increased production of anti-inflammatory cytokines (IL-10 and TGFβ1) and decreased production of the proinflammatory cytokine, TNFα. Ultimately, this altered cytokine profile drives PTB progression by compromising host resistance to Mtb.

This study used MR for causal inference, single-cell and bulk multi-omics analyses, and experimental validation to systematically address the following key questions: 1) is there a causal association between serum caffeine levels and PTB immunosuppression?, 2) if yes, what are the core effector immune cells and molecular mechanisms involved?, and 3) can PTB-associated biomarkers with translational value be identified based on this mediating MR analysis? Our study provides the first evidence that excessive serum caffeine level may promote PTB immunosuppression by activating CD39^+^CD4^+^ Tregs. This process relies on the CD39-mediated enhancement of adenosine receptor binding capacity, which is primarily achieved through the PSMC5, BAG1, and AGPAT5 proteins (**Figure 9**).

This study demonstrates innovation and reliability in establishing a causal relationship between excessive serum caffeine levels and PTB immunosuppression. This is consistent with previous Mendelian randomization studies showing that caffeine and coffee consumption correlates with multiple adverse health outcomes (6, 7). In lung tissue-related diseases, elevated plasma caffeine levels are associated with a 54% increased risk of lung cancer based on a MR study (22), and a meta-analysis has shown that each additional cup of coffee consumed per day is associated with a 6% higher risk of lung cancer (23). Because previous studies of the association between caffeine and infectious diseases have predominantly been observational, it is difficult to establish causality because they were susceptible to lifestyle confounding factors, such as smoking and dietary patterns (24, 25). Through TSMR analysis of 486 blood metabolites, this study identified excessive serum caffeine levels may as a risk factor for PTB immunosuppression. Mediation analysis integrating 731 immune cell types confirmed CD39^+^CD4^+^ Tregs as the main mediator of this association. By excluding reverse causality, sensitivity and reverse MR analyses collectively validated the robustness of this causal relationship. We provide genetic evidence from genome-wide association study (GWAS) for the association between serum caffeine levels and PTB risk. Additionally, we systematically revealed the specific mediating role of CD4^+^ Tregs. We divided CD4^+^ Tregs into five subsets, including CD39^+^CD73^-^ and CD39^+^CD73^+^, and identified CD39^+^CD4^+^ Tregs as the only subset associated with the protective effect of serum caffeine levels against PTB risk. In contrast, the CD39^-^ Tregs subset showed no significant association. This finding suggests CD39 as a key molecular switch in the regulation of Treg function in response to caffeine. It also suggests that previous studies failed to identify a clear association between Tregs and caffeine because they overlooked Treg heterogeneity (26).

Caffeine activates CD39^+^CD4^+^ Tregs via CD39 upregulation and enhanced adenosine receptor binding capacity. In high caffeine response Tregs, CD39 and A2A-R were co-elevated, with a significantly enhanced adenosine receptor binding capacity. Additionally, caffeine significantly increases the proportion of CD39^+^ Tregs and the expression of adenosine receptor-binding-related proteins in the lungs of Mtb-infected mice. These suggest that the direct binding of caffeine to the A2A-R (27). is masked by the resulting accumulation of adenosine in PTB. Specifically, although caffeine directly binding to A2A-R, it promotes extracellular ATP hydrolysis to adenosine by upregulating CD39. High adenosine concentrations initiate downstream immunosuppressive pathways by competitively activating the A2A-R. This mechanism explains the discrepancy between *in vitro* and *in vivo* results and enriches our understanding of the molecular network governing the regulation of Treg activity by caffeine. Moreover, it establishes CD39-mediated adenosine concentration amplification as the pivotal link connecting *in vitro* receptor antagonism with *in vivo* immunosuppressive effects. Transcriptomic analysis and GSEA revealed enrichment for the “protein folding pathway”, e.g., the elevated expression of the transcription factors, HSF1 and SRF, in CD39^+^ Tregs with high adenosine receptor binding capacity. In contrast, “immune cell differentiation” and “G protein-coupled receptor signaling pathways” were suppressed, which is consistent with previous findings (28–30). This suggests that caffeine-activated CD39^+^ Tregs may enhance immunosuppression by maintaining protein homeostasis but inhibit differentiation into effector T cells, thereby establishing “immune tolerance” in the Mtb infection microenvironment and promoting pathogen colonization. Caffeine increased Treg and CD39^+^ Treg in non-PTB mice, showing disease-independent Treg regulation. Our animal experiments revealed that the caffeine-treated groups exhibited worse lung tissue damage and inflammation compared to the infection control group.

Based on the mechanism by which caffeine activates Tregs via the CD39-adenosine pathway, we used a multidimensional data integration strategy to identify translatable molecular biomarkers. The integrated approach mitigated technical noise in single-cell data, while overcoming cellular contamination in RNA-seq data. ROC curve analyses revealed that PSMC5, BAG1, and AGPAT5, which, based on scRNA-seq/bulk RNA-seq integration and LASSO regression analysis, were differentially expressed in PTB versus normal tissue (*P <* 0.05), demonstrated a certain level of diagnostic performance (AUC > 0.7). Compared with traditional PTB diagnostic markers, these biomarkers reflect host immune status and may be used for early PTB diagnosis or the assessment of treatment efficacy. Moreover, the caffeine monomer upregulated PSMC5, BAG1, and AGPAT5 expression in Mtb-infected THP-1 macrophages and mouse lung tissue. This suggests these genes as downstream effector molecules of the CD39–adenosine pathway, highlighting them as candidates for the development of targeted therapeutics, especially PSMC5.

This study has the following advantages over previous studies. While previous studies primarily examined the overall role of Tregs in PTB (31), ours defines the “caffeine-responsive CD39^+^ Treg” subset, clarifies its differentiation trajectory, molecular characteristics, and functional effects, and offers a new perspective on understanding the complexity of the PTB immune microenvironment. Caffeine often exhibits protective effects against metabolic diseases (32, 33), our study reveals it increases immunosuppression via CD39^+^ Tregs, suggesting that the immunological effects of serum caffeine are “disease-specific”. This discovery provides an immunological basis for personalized health risk assessment based on serum caffeine levels. Although the role of the CD39–adenosine pathway in immunosuppression is well defined (34, 35), its therapeutic potential in PTB is unexplored. Our study confirmed that this pathway is central to the regulation of PTB risk by caffeine and provides comprehensive evidence for the development of CD39 or A2A-R inhibitors as host-directed therapeutics for PTB.

This study has some limitations. For MR analysis, we used GWAS data from European populations. However, our MR results are highly robust: IVs were selected with strict criteria (LD r² < 0.001, F > 10) to avoid weak instrument bias, and sensitivity analyses (leave-one-out, MR-Egger intercept, Cochran’s Q test) confirmed no single SNP-driven effect (Δβ < 0.15) or horizontal pleiotropy (all *P* > 0.05). To address this limitation, a causal relationship between serum caffeine levels and PTB in Asian and African populations requires further evaluation. A key limitation is that whole-lung bulk qPCR cannot definitively link changes in PSMC5, BAG1, and AGPAT5 expression to CD4^+^ Tregs, even though our scRNA-seq and mIHC data support their cell-type-specific changes in this population. Future studies will be needed to explore the molecular mechanisms by which PSMC5 regulates the CD39–adenosine pathway in CD4^+^ Tregs, macrophages, and B cells. Third, we did not explore the dose-dependent immunomodulatory effects of caffeine in our experimental system and direct plasma caffeine and antitubercular cytokines such as IFN-γ level. Therefore, the dose-response relationship of caffeine on PTB progression, and its differential regulatory effects on various immune cell subsets, should be systematically evaluated in future preclinical and clinical studies. Additionally, future studies should consider conducting multiethnic GWAS meta-analyses to clarify population heterogeneity in the excessive serum caffeine level–PTB immunosuppression association. Furthermore, the current study has not verified the direct causal role of CD39^+^ Tregs in caffeine-promoted PTB progression via Treg-specific depletion animal experiments. This specific causal effect needs to be further confirmed by cell-specific loss-of-function experiments in future.

## 5 Conclusion

We for the first time establish a causal link between serum caffeine levels, CD39^+^CD4^+^ Tregs, the CD39^-^ adenosine pathway, and PTB immunosuppression, and identify core biomarkers with translational value. Our findings provide a novel immunometabolic perspective on PTB pathogenesis and offer a foundation for the development of precise diagnostic tools and host-directed therapeutic strategies. They also highlight strategies for the exploration of the association between plasma metabolites and infectious diseases.

## Data Availability

The code involved in this study can be obtained from the first author. Single cell RNA-seq datasets used in this study were available the National Center for Biotechnology Information (NCBI) Gene Expression Omnibus (https://www.ncbi.nlm.nih.gov/geo) under accession number GSE192483, GSE148036, and GSE114911.
