# Supplementary material for: Caffeine-mediated CD39+ Treg activation via the CD39-adenosine receptor pathway is a novel risk factor for pulmonary tuberculosis
Source: Front Immunol. 2026 Apr 20;17:1784235. doi: 10.3389/fimmu.2026.1784235 (PMC13135997; doi:10.3389/fimmu.2026.1784235)
Supplement: Supplementary file 1 [file Image1.pdf]

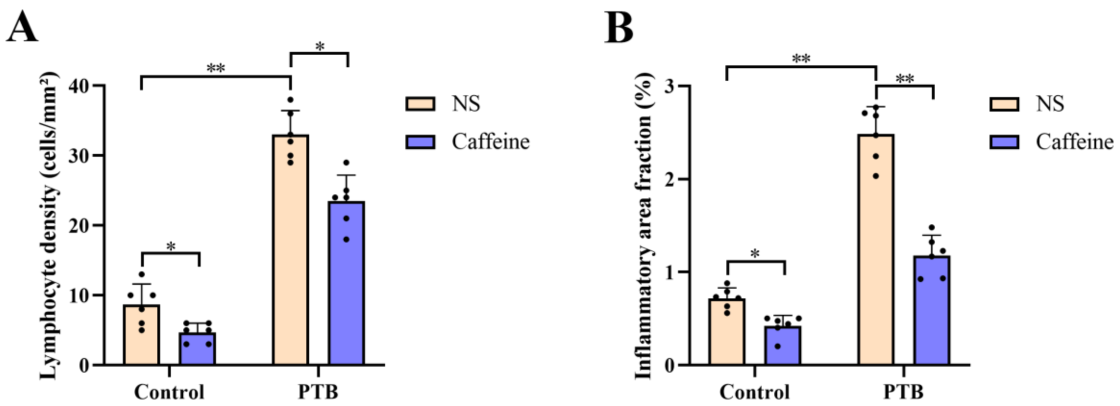

Supplementary Figure S1. Caffeine reduces pulmonary lymphocyte infiltration and inflammatory lesion area in the PTB model mice. A Quantitative analysis of lung lymphocyte density (cells/mm²) in Control and PTB mice with or without caffeine intervention. **B** Quantitative analysis of inflammatory area fraction (%) in lung tissue sections. Data are presented as mean ± SD (n=6 mice per group). *, and ** indicate *P* < 0.05, and < 0.01.

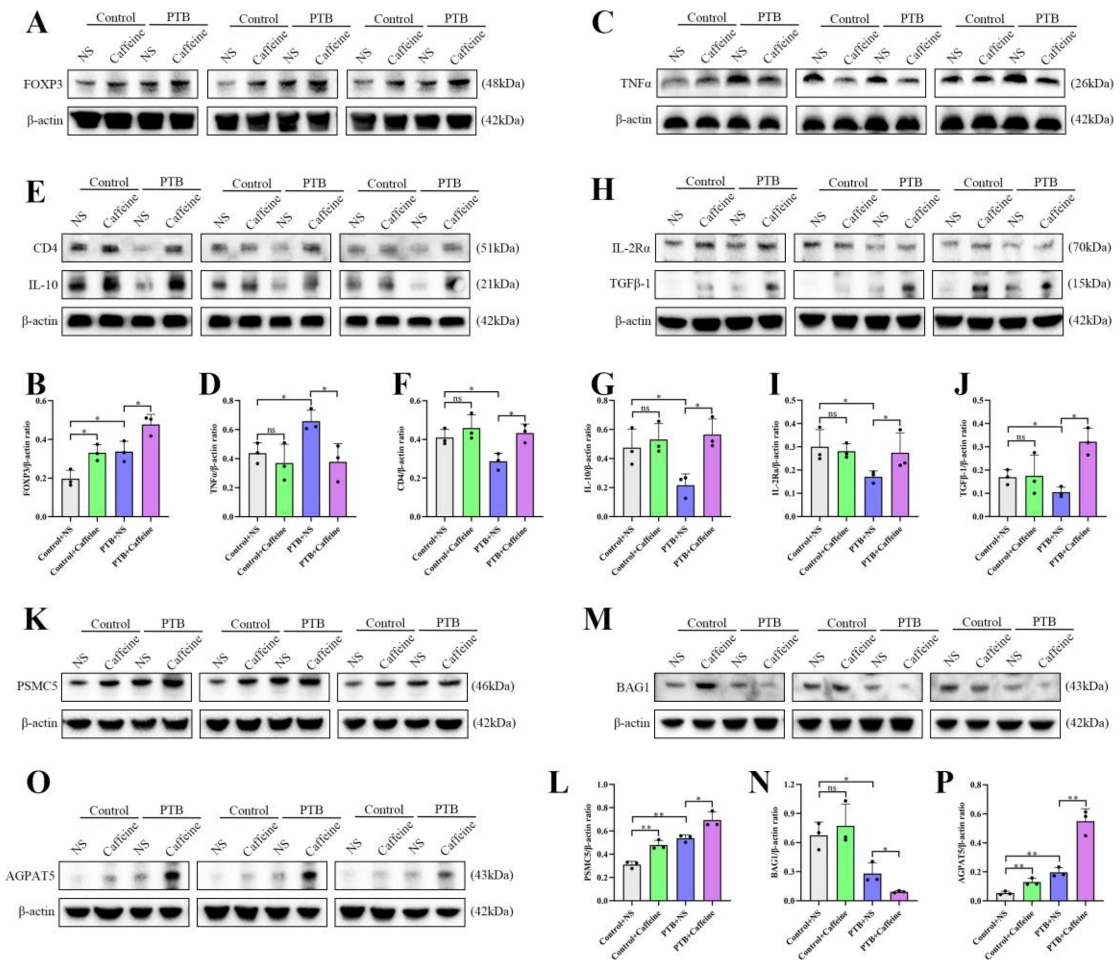

Supplementary Figure S2. WB validation of target protein expression in PTB mice with caffeine intervention. Paired representative WB bands and quantitative analysis of target proteins in lung tissues from 4 experimental groups (Control+NS, Control+Caffeine, PTB+NS, PTB+Caffeine, n=3 biologically independent samples per group): **(A, B)** FOXP3; **(C, D)** TNF-α; **(E-G)** CD4 and IL-10; **(H-J)** IL-2Rα and TGF-β1; **(K, L)** PSMC5; **(M, N)** BAG1; **(O, P)** AGPAT5. β-actin was used as the endogenous loading control. Protein expression was quantified by normalizing band gray intensity to the paired β-actin from the same sample. Data are presented as mean ± SD. ns, *, and ** indicate not significant, $P < 0.05$, and < 0.01.

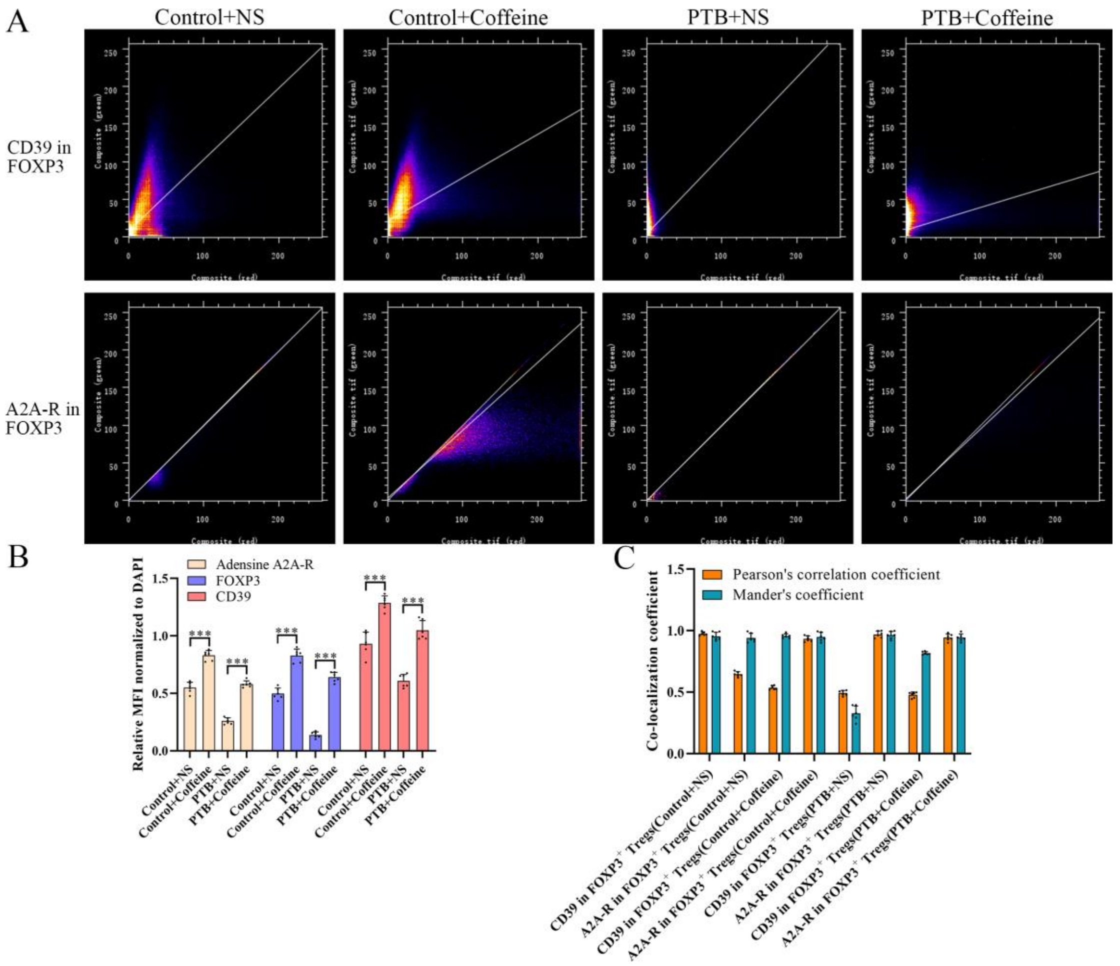

Supplementary Figure S3. MIHC staining, quantitative expression analysis, and co-localization verification of CD39, Adenosine A2A-R, and FOXP3 in mouse lung tissues. A Dual-channel fluorescence intensity scatter plot of co-localization analysis. The upper row shows the signal distribution of CD39 (x-axis) and FOXP3 (y-axis), and the lower row shows the signal distribution of Adenosine A2A-R (x-axis) and FOXP3 (y-axis) in lung tissues. The white diagonal line represents the reference line for complete signal co-localization. **B** Quantitative analysis of the relative mean fluorescence intensity (MFI) of Adenosine A2A-R, FOXP3, and CD39. MFI was normalized to the corresponding DAPI nuclear signal to eliminate section-to-section and imaging variation, with the mean value of the Control+NS group set to 1. **C** Quantitative co-localization coefficients between target proteins (CD39/Adenosine A2A-R) and the Treg-specific marker FOXP3. Pearson's correlation coefficient (orange) reflects the linear correlation of signal intensity between two channels; Mander's overlap coefficient (blue). All analyses were performed by two independent investigators in a blinded manner, n=6 biologically independent mice per group. *** indicate $P < 0.001$.

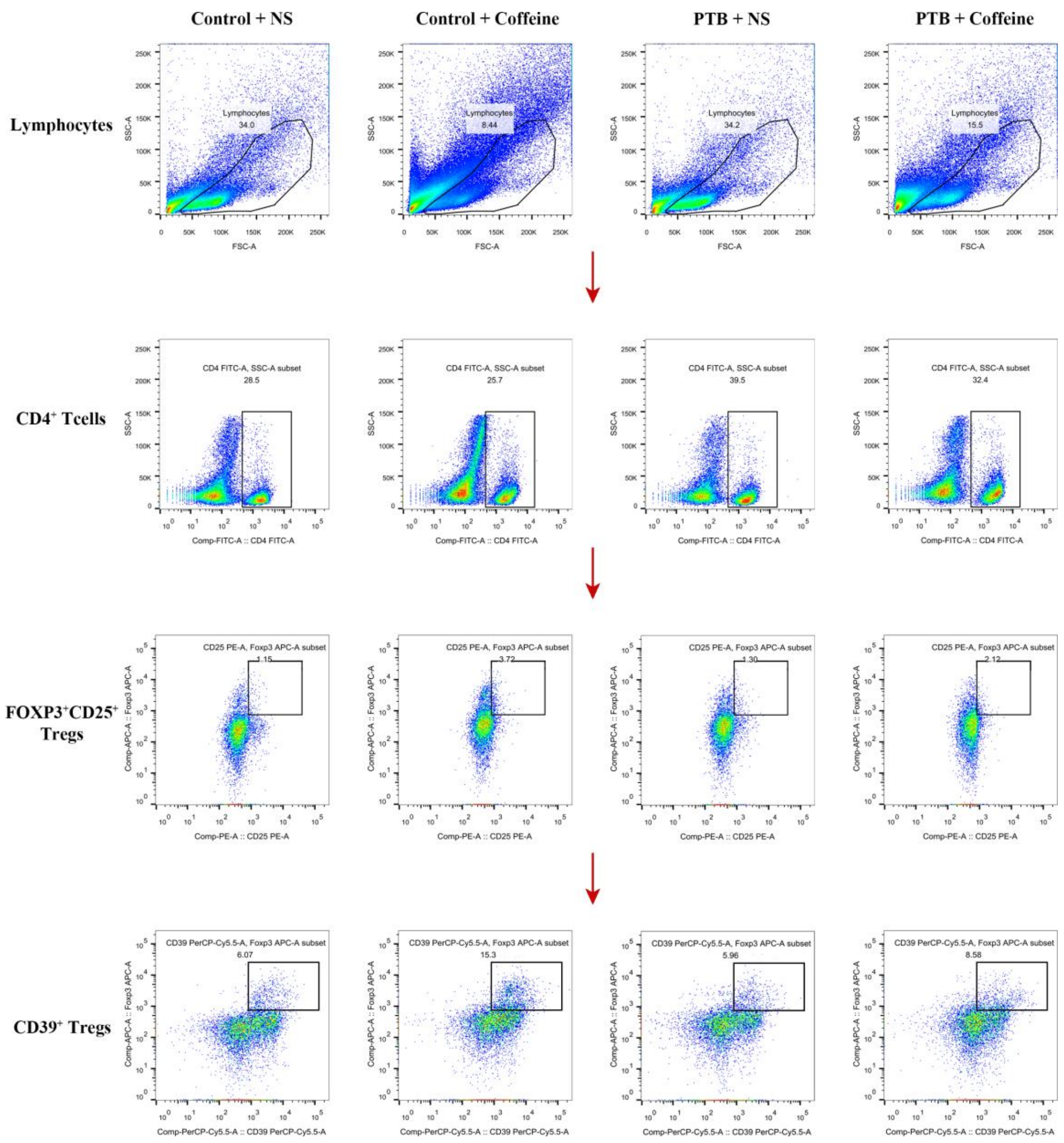

Supplementary Figure S4. Gating strategy for identification of CD39$^+$CD4$^+$FOXP3$^+$CD25$^+$ Tregs. Flow cytometry plots showing the sequential gating strategy: (1) FSC-A vs SSC-A to gate lymphocytes; (2) CD4 vs SSC-A to identify CD4$^+$ T cells; (3) FOXP3 vs CD25 to define FOXP3$^+$CD25$^+$ Tregs; (4) CD39 expression within FOXP3$^+$ Tregs to quantify CD39$^+$ Treg subset.

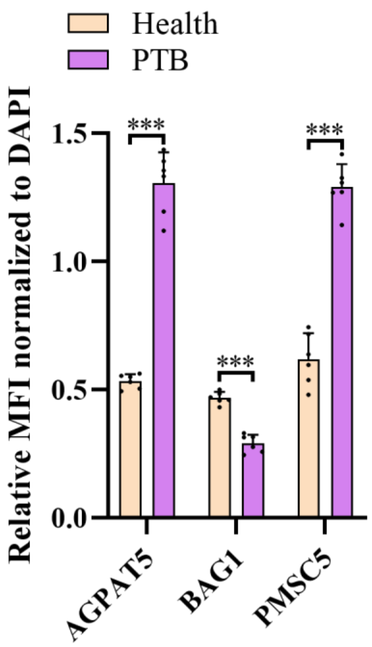

Supplementary Figure S5. Quantitative analysis of core adenosine pathway marker expression in lung tissues from PTB patients and healthy controls. Relative MFI of AGPAT5, BAG1, and PMSC5 in lung tissue sections from healthy controls and patients with PTB, normalized to DAPI nuclear staining. Data are presented as mean ± SD (n=6 independent samples per group). *** indicate $P < 0.001$.

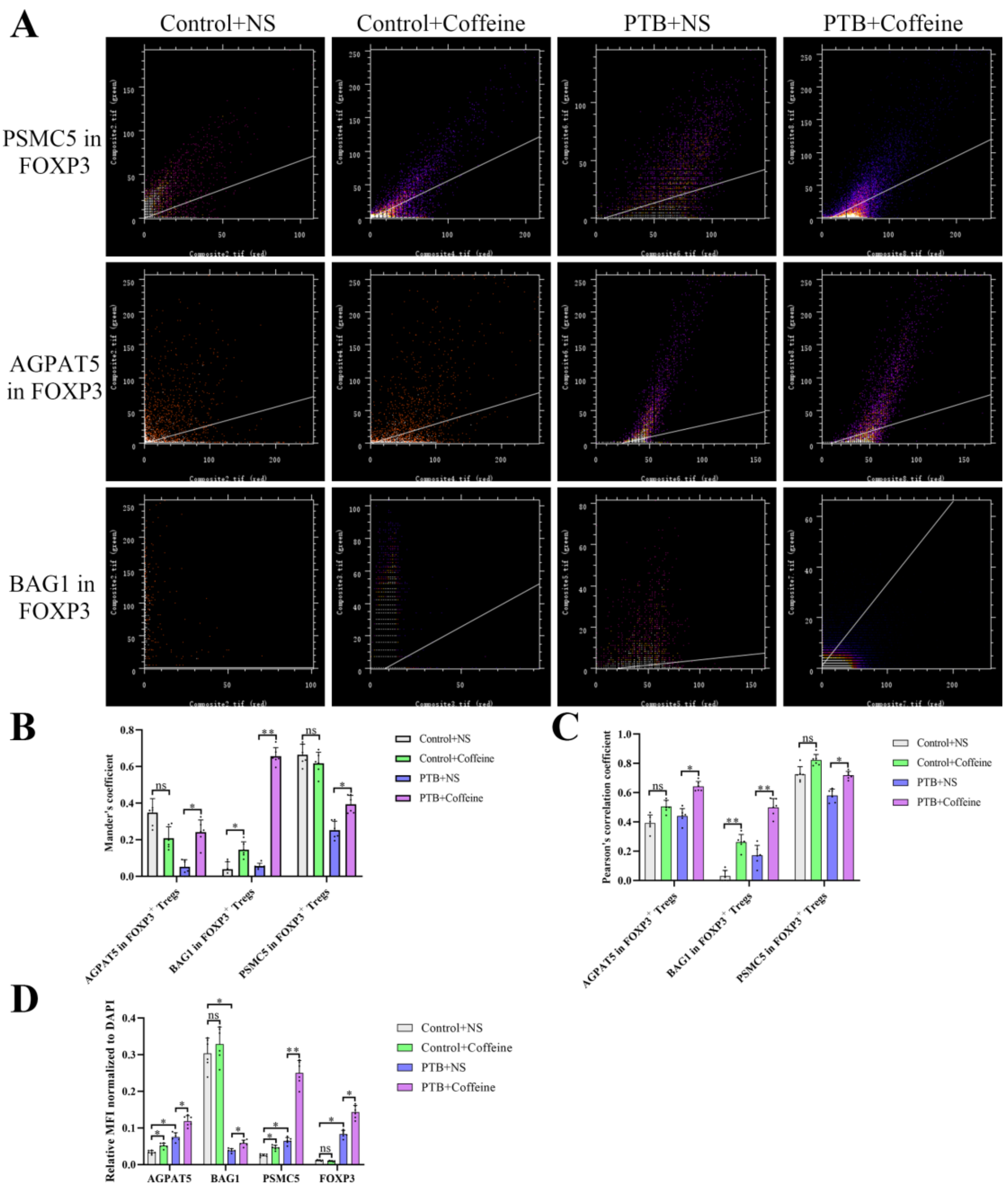

Supplementary Figure S6. Caffeine modulates the colocalization and expression of core adenosine pathway markers in FOXP3^{+} Tregs in the murine PTB model. A Representative scatter plots of colocalization between core markers (AGPAT5, BAG1, PSMC5) and FOXP3^{+} Tregs in lung tissues from 4 groups (Control+NS, Control+Caffeine, PTB+NS, PTB+Caffeine). **B** Quantitative analysis of Manders' colocalization coefficients for each marker in FOXP3^{+} Tregs. **C** Quantitative analysis of Pearson's correlation coefficients for each marker in FOXP3^{+} Tregs. **D** Relative MFI of target proteins normalized to DAPI. Data are presented as mean ± SD (n=6 mice per group). ns, *, and ** indicate not significant, $P < 0.05$, and < 0.01.
